# Effects of joint screening for prostate, lung, colorectal, and ovarian cancer – results from a controlled trial

**DOI:** 10.3389/fonc.2024.1322044

**Published:** 2024-04-29

**Authors:** Zeyu Fan, Yu Zhang, Qiaoling Yao, Xiaomin Liu, Hongyuan Duan, Ya Liu, Chao Sheng, Zhangyan Lyu, Lei Yang, Fangfang Song, Yubei Huang, Fengju Song

**Affiliations:** ^1^ Department of Epidemiology and Biostatistics, Key Laboratory of Molecular Cancer Epidemiology, Tianjin, National Clinical Research Center for Cancer, Tianjin Medical University Cancer Institute and Hospital, Tianjin Medical University, Tianjin, China; ^2^ Key Laboratory of Carcinogenesis and Translational Research (Ministry of Education), Beijing Office for Cancer Prevention and Control, Peking University Cancer Hospital & Institute, Beijing, China

**Keywords:** combined risk assessment, risk stratification, screening effectiveness, screening compliance, PLCO cancer, joint cancer screening

## Abstract

**Background:**

Although screening is widely used to reduce cancer burden, untargeted cancers are frequently missed after single cancer screening. Joint cancer screening is presumed as a more effective strategy to reduce overall cancer burden.

**Methods:**

Gender-specific screening effects on PLCO cancer incidence, PLCO cancer mortality, all-neoplasms mortality and all-cause mortality were evaluated, and meta-analyses based on gender-specific screening effects were conducted to achieve the pooled effects. The cut-off value of time-dependent receiver-operating-characteristic curve of 10-year combined PLCO cancer risk was used to reclassify participants into low- and high-risk subgroups. Further analyses were conducted to investigate screening effects stratified by risk groups and screening compliance.

**Results:**

After a median follow-up of 10.48 years for incidence and 16.85 years for mortality, a total of 5,506 PLCO cancer cases, 1,845 PLCO cancer deaths, 3,970 all-neoplasms deaths, and 14,221 all-cause deaths were documented in the screening arm, while 6,261, 2,417, 5,091, and 18,516 outcome-specific events in the control arm. Joint cancer screening did not significantly reduce PLCO cancer incidence, but significantly reduced male-specific PLCO cancer mortality (hazard ratio and 95% confidence intervals [HR(95%CIs)]: 0.88(0.82, 0.95)) and pooled mortality [0.89(0.84, 0.95)]. More importantly, joint cancer screening significantly reduced both gender-specific all-neoplasm mortality [0.91(0.86, 0.96) for males, 0.91(0.85, 0.98) for females, and 0.91(0.87, 0.95) for meta-analyses] and all-cause mortality [0.90(0.88, 0.93) for male, 0.88(0.85, 0.92) for female, and 0.89(0.87, 0.91) for meta-analyses]. Further analyses showed decreased risks of all-neoplasm mortality was observed with good compliance [0.72(0.67, 0.77) for male and 0.72(0.65, 0.80) for female] and increased risks with poor compliance [1.61(1.40, 1.85) for male and 1.30(1.13, 1.40) for female].

**Conclusion:**

Joint cancer screening could be recommended as a potentially strategy to reduce the overall cancer burden. More compliance, more benefits. However, organizing a joint cancer screening not only requires more ingenious design, but also needs more attentions to the potential harms.

**Trial registration:**

NCT00002540 (Prostate), NCT01696968 (Lung), NCT01696981 (Colorectal), NCT01696994 (Ovarian).

## Introduction

Cancer ranks as the second leading cause of death and the primary cause of years of life lost, years lived with disability and disability-adjusted life-years in several countries around the world (1–3). An estimated 19.3 million new cancer cases and almost 10 million cancer deaths occurred in 2020 (1). Due to the rapidly growing epidemic of several cancer-associated risk factors, including aging, tobacco use, unhealthy diet, excess body weight, physical inactivity, and air pollution, the cancer burden is expected to grow in most parts of the world, particularly in transitioning or developing countries (1, 4, 5). Addressing the growing global burden of cancer has become a global concern.

For more than a half of century, cancer screening has been widely used as an effective strategy to reduce the cancer burden in several countries. Until now, several high-level evidences had supported that population-based screening for cancer at different sites (including colorectum, cervix uteri, lung, breast, and prostate) can reduce cancer-specific mortality by about 18%-20% (6–12), and similar conclusions can be observed across different periods (13–17). Even in countries with limited resources, one-time population-based mass screening has also been shown to be associated with a significant reduction in cancer-specific incidence and mortality (18–20). Other research efforts are also underway to reduce the burden of cancers with relatively low incidence and poor prognoses, such as ovarian cancer and liver cancer (21, 22). However, in both well-resourced and resource-limited areas, previous and current cancer screening programs just focus on one site of cancer at a time (6–12, 23, 24). Untargeted cancers are often missed and later reported after a single cancer screening. To reduce the burden of cancer uncovered by the previous screening program, policymakers have to organize another independent screening program for uncovered cancer. When this demand involves several cancers at multiple sites, duplication of investment and waste of limited resources will be inevitable in the context of a single cancer screening strategy. Therefore, a new screening mode, namely joint cancer screening, has been raised as a more effective screening strategy to reduce the overall cancer burden than the current single cancer screening strategy (25). Joint cancer screening means that individuals in the screening group received multiple methods recommended by the guidelines to screen for different cancers during the same period. More importantly, risk-stratified joint cancer screening would be a more cost affordable or sustainable screening strategy for regions or countries with large populations but limited resources. However, few studies have investigated the effect of joint cancer screening, and fewer studies have investigated the effect of risk-stratified joint cancer screening.

The Prostate, Lung, Colorectal, and Ovarian (PLCO) Cancer Screening Trial is currently the only published randomized, controlled trial (RCT) in the world to screen prostate, lung, colorectal and ovarian cancer concurrently in the same population (26). The study had previously reported the screening effectiveness for four targeted cancers (9, 27–29). However, no study has evaluated the effectiveness of joint cancer screening based on its exclusive “all-versus-none” design, especially the effectiveness of risk-stratified joint cancer screening based on additional follow-up and outcomes. Therefore, this study aims to investigate the effectiveness of joint cancer screening on PLCO cancer incidence, PLCO cancer mortality, all-neoplasms mortality, and all-cause mortality, and try to develop a risk-stratified joint cancer screening strategy suitable for countries with increasing cancer burden and limited resources.

## Method

### Source of population and selection of participants

The design and protocol of the PLCO cancer screening trial have been described previously (30). Briefly, the PLCO cancer screening trial is an RCT that enrolled 155,000 participants aged 55 to 74 between November 1993 and July 2001. Participants were individually randomized to the control arm or intervention arm in equal proportions. Participants assigned to the control arm received usual care, whereas participants assigned to the intervention arm received screening exams for prostate, lung, colorectal, and ovarian cancers as outlined in the study protocol. Men and women received organ-specific screening protocols, respectively. Men received a combination of a six-round annual prostate-specific antigen (PSA) exam and a four-round annual digital rectal examination (DRE) to detect prostate cancer. Women received a combination of six-round annual cancer antigen 125 (CA-125) and four-round annual transvaginal ultrasound (TVU) to detect ovarian cancer. Both men and women were given a four-round annual posteroanterior chest X-ray (XRY) to detect lung cancer and two-round flexible sigmoidoscopy (FSG) with an interval of three to five years to detect colorectal cancer.

In this study, a total of 149,161 eligible participants were initially included after excluding 4,918 participants with no baseline questionnaire returned, 185 participants with a history of PLCO cancer, and 623 participants lost to follow-up. After further excluding 10,322 participants with missing data of index predictors of PLCO cancer and 6,778 participants with a history of any other cancers, a total of 66,490 male participants and 65,533 female participants were selected for further analyses. In the screening arm, there were 1,635 male participants and 2,619 female participants who did not receive any screening, these individuals who were non-compliant with screening were compared to those who were compliant with screening in sensitivity analyses based on screening compliance. Furthermore, after excluding participants who did not received at least one-round joint cancer screening, 61,248 male participants and 57,240 female participants were finally included in the preliminary analyses. Detailed flowchart referred to **Supplementary Figure 1**.

### Positive screening exams and diagnostic evaluation

As defined by the PLCO protocol (9, 26–29), the criteria for a positive screening exam included as follows: 1) XRY, one or more nodules, mass, hilar or mediastinal lymph node enlargement, infiltrate, consolidation, or alveolar opacity. 2) FSG, one or more rectal nodules, rectal and/or colon mass, colon polyp. 3) PSA, > 4 ng/ml. 4) DRE, one or more nodularity, induration, asymmetry, and loss of anatomic landmarks. 5) CA-125, ≥ 35 U/ml. 6) TVU, one or more ovary or cyst > 10 cc; solid area, or papillary projection extending into the cavity of a cystic ovarian tumor of any size; mixed (solid/cystic) component within a cystic ovarian tumor.

Participants who had received a positive screening result were encouraged to receive a further diagnostic evaluation. Participants and their physicians decided which procedures would be performed. In the case of a positive FSG, it was standard practice not to biopsy or remove polyps or masses; instead, a referral was made to the participant’s physician for diagnostic evaluation and follow-up.

### Cancer diagnosis and primary outcomes

The PLCO trial confirmed diagnoses of cancer through medical record abstraction (MRA), which was triggered by (9, 26–29): 1) self-report of cancer on an Annual Study Update (ASU); 2) positive screening exams mentioned above; 3) death certificate indicating cancer; 4) Death Review Process (DRP) suspected cancer based on other indicators; 5) relative informs screening center of participant’s cancer. Cancer diagnosis was followed up to December 31, 2009 and mortality was followed up to December 31 2018. For every cancer that was ascertained, information on the cancer diagnosis date and ICD-O-2 code was collected and recorded on the appropriate medical record abstract form. For primary PLCO cancer ascertained, additional information on diagnostic procedures, cancer stage, grade, histopathologic type, and initial cancer treatment was further recorded.

The primary outcomes of this study included PLCO cancers incidence, PLCO cancers mortality, all-neoplasms mortality and all-cause mortality. To assess these endpoints, all deaths from any cause were collected and ascertained according to the following main sources: 1) the administration of the ASU questionnaires, 2) reports from relatives, friends, or physicians, and 3) National Death Index (NDI) plus searches. After notification, PLCO Screening Centers attempted to obtain a death certificate for each death before 2018. The underlying leading cause of death was derived according to rules established by the National Center for Health Statistics. To provide a more accurate assessment of the trial endpoints, a DRP was conducted and medical records were reviewed for all deaths that might have been due to PLCO cancers. The DRP cause of death was considered authoritative and was used in statistical analyses of the primary endpoints.

### Assessment of covariates

After informed consent, all participants were provided with a baseline questionnaire to collect participant-reported information on demographic and potential risk factors associated with PLCO cancers, including age, sex, race, height, weight, smoking status, medical history (including diabetes, hypertension, heart attack, stroke, bronchitis, emphysema, liver comorbidity, colon comorbidity, polyps, diverticulitis), regular use of non-steroidal anti-inflammatory drugs (NSAIDs) (including aspirin and ibuprofen) in the last 12 months, sex-specific information (such as oral contraceptives and hormone replacement therapy for female, and enlarged prostate, prostatitis, and prostate surgery for male), personal history of cancer (including PLCO cancers and any other cancers), personal screening history of PLCO cancer, and family history of any cancer among first-degree relative. Body mass index (BMI) was calculated as weight in kilograms divided by the square of height in meters (kg/m^2^).

### Statistical analysis

Chi-square tests were used to compare the distribution of potential confounding factors associated with screening effectiveness between the screening arm and control arm. Gender-specific cumulative PLCO cancer incidence, PLCO cancer mortality, all-neoplasms mortality, and all-cause mortality were calculated with Kaplan-Meier curves and compared with log-rank tests. Gender-specific screening effectiveness of primary endpoints was evaluated with multivariable Cox regression analyses after adjusting all available confounding factors. Due to different gender-specific factors associated with primary outcomes, meta-analyses based on gender-specific screening effectiveness rather than multivariable Cox regression analyses adjusting gender were conducted to achieve the pooled effectiveness. The I² statistic was calculated to determine the heterogeneity between gender-specific screening effectiveness (31). Subgroup analyses based on combined PLCO cancer risk assessment and sensitivity analyses by screening compliance were further conducted to explore the effectiveness of risk-stratified joint cancer screening.

To develop a gender-specific PLCO cancer risk prediction model (PLCO-CA model) for the general population without intervention, 70% of the participants in the control arm were randomly assigned to the derivation cohort (22,726 male participants and 22,798 female participants), while the remaining 30% were assigned to the validation cohort (9,778 male participants and 9,772 female participants). Since diabetes has been extensively studied concerning the increased risk of developing cancer (32), diabetes has been listed as the potentially independent predictor of PLCO cancer. History of organ-related partial resection surgery would suggest a greater potential risk of cancer than other benign organ-related diseases, so it was also used as a potentially independent predictor of PLCO cancer risk. To achieve relatively stable and easy-to-use sex-specific PLCO-CA models, chronic bronchitis, emphysema, polyps, diverticulitis or diverticulosis, colon comorbidity, prostatitis (male), enlarged prostate or benign prostatic hypertrophy (male), and benign ovarian tumor/cyst (female) were combined and defined as organ-related diseases (0, 1, ≥2). Stroke, heart attack, hypertension, and liver comorbidity were combined and defined as non-organ-related diseases (0, 1, ≥2). All available PLCO cancer-associated sex-specific predictors were listed in **Supplementary Tables 1**, **2**, and they were initially selected to develop sex-specific PLCO-CA models.

In the derivation cohort, univariate Cox regression was first used to test the crude association of PLCO cancer with each potential predictor. The potential predictors with P < 0.25 were preliminarily selected into multivariable Cox regression. P value < 0.05 for entry and P value > 0.10 for removal were used in the both forward and backward variable selection procedures within the multivariable Cox regression to select independent predictors of PLCO cancers. The hazard ratios (HR) and its 95% confidential interval (CI) were calculated to measure the association between predictor and PLCO cancer risk. The discriminations of the gender-specific PLCO-CA models were evaluated by the receiver-operating-characteristic curves (ROCs) and the areas under the curve (AUCs). The calibrations of PLCO-CA models were assessed by calibration curves and measured as the ratio of the observed and expected number of cases (O/E ratio). The external validations of PLCO-CA models were also measured as the AUCs and the O/E ratios of these models in the validation cohort. The optimal cut-off value of time-dependent ROC of 10-year PLCO cancer risk in the derivation cohort was used to classify participants into low- and high-risk groups.

Additionally, previous studies suggested that PLCO cancer screening can only significantly reduce the specific mortality of colorectal cancer (CRC) but not for other three cancers (9, 27–29). To avoid the driving effect of CRC screening on joint cancer screening, further CRC-free sensitivity analyses were conducted to reassess the effects of joint cancer screening on all primary outcomes after excluding CRC cases (N=1656) and deaths (N=671) censored at the date of CRC incidence or mortality.

All statistical analyses were conducted via R software (version 4.1.0). The ‘survival’ package (version 3.3-1) was used to conducted survival analyses. The ‘survminer’ package (version 0.4.9) was used to draw survival curves. The ‘survivalROC’ package (version 1.0.3.1) was used to draw time-dependent ROC curve and calculate the area under ROC curve. The ‘meta’ package (version 5.5-0) was used to conduct meta analyses. The ‘ggplot2’ package (version 3.3.5) was used to draw the cancer stage graphs, and forest plots were drawn with ‘forestplot’ package (version 2.0.1). P value < 0.05 was considered statistically significant.

## Result

### Baseline characteristics of participants between the screening arm and control arm

As shown in **Table 1**, in both males and females, compared with the control arm, the screening arm seemed to have younger age, fewer smokers, and fewer history of organ-related partial resection surgery. Some other differences can also be found between the gender-specific screening and control arms. For example, male participants in the screening arm had more family history of cancer compared to their counterparts in the control arm, while female participants in the screening arm used more hormones for menopause. However, none of the absolute differences in subgroup-specific proportions within each index variable between the screening arm and control arm was greater than 2%. Therefore, these differences could only be considered as statistical differences related with the specific selection flowchart rather than actually differences in baseline characteristics between the two arms.

**Table 1:** Comparison of baseline characteristics between the screening arm and the control arm by gender.

Characteristics	Subgroups	Male, n (%)			Female, n (%)		
		Control arm	Screening arm	P value	Control arm	Screening arm	P value
		(N=32,466)	(N=28,802)		(N=32,570)	(N=24,670)	
Age, years	55-59	10383(31.98%)	9529(33.08%)	<0.001	11232(34.49%)	8761(35.51%)	<0.001
	60-64	10257(31.59%)	9176(31.86%)		9899(30.39%)	7732(31.34%)	
	65-69	7530(23.19%)	6556(22.76%)		7123(21.87%)	5255(21.30%)	
	70-74	4296(13.23%)	3541(12.29%)		4316(13.25%)	2922(11.84%)	
Race	White	28747(88.54%)	25639(89.02%)	0.066	28924(88.81%)	21933(88.91%)	0.717
	Non-white	3719(11.46%)	3163(10.98%)		3646(11.19%)	2737(11.09%)	
Smoking	Never	11821(36.41%)	10942(37.99%)	<0.001	18270(56.09%)	14220(57.64%)	<0.001
	Current	3825(11.78%)	3133(10.88%)		3099(9.51%)	2119(8.59%)	
	Former	16820(51.81%)	14727(51.13%)		11201(34.39%)	8331(33.77%)	
BMI, kg/m^2^	<25	8785(27.06%)	7654(26.57%)	0.126	13478(41.38%)	10069(40.81%)	0.261
	25-29.9	16428(50.60%)	14530(50.45%)		11254(34.55%)	8535(34.60%)	
	≥30	7253(22.34%)	6618(22.98%)		7838(24.07%)	6066(24.59%)	
Aspirin user	No	15677(48.29%)	13932(48.37%)	0.841	18828(57.81%)	14276(57.87%)	0.892
	Yes	16789(51.71%)	14870(51.63%)		13742(42.19%)	10394(42.13%)	
Ibuprofen user	No	25087(77.27%)	22101(76.73%)	0.117	21948(67.39%)	16638(67.44%)	0.896
	Yes	7379(22.73%)	6701(23.27%)		10622(32.61%)	8032(32.56%)	
Diabetes	No	29613(91.21%)	26377(91.58%)	0.108	30513(93.68%)	23219(94.12%)	0.033
	Yes	2853(8.79%)	2425(8.42%)		2057(6.32%)	1451(5.88%)	
Prostate (male)/	None	29797(91.78%)	26797(93.04%)	<0.001	26066(80.03%)	20071(81.36%)	<0.001
Ovary (female)	Fully removed	1424(4.39%)	918(3.19%)		4104(12.60%)	2950(11.96%)	
surgery	Other	1245(3.83%)	1087(3.77%)		2400(7.37%)	1649(6.68%)	
Organ-related disease	0	20927(64.46%)	18780(65.20%)	0.136	23307(71.56%)	18083(73.30%)	<0.001
	1	8042(24.77%)	7017(24.36%)		7398(22.71%)	5309(21.52%)	
	≥2	3497(10.77%)	3005(10.43%)		1865(5.73%)	1278(5.18%)	
Non-organ-related disease	0	18297(56.36%)	16788(58.29%)	<0.001	20184(61.97%)	15590(63.19%)	0.001
	1	10974(33.80%)	9486(32.94%)		10761(33.04%)	7976(32.33%)	
	≥2	3195(9.84%)	2528(8.78%)		1625(4.99%)	1104(4.48%)	
Screening history	No	5982(18.43%)	5635(19.56%)	<0.001	9114(27.98%)	7071(28.66%)	0.075
	Yes	26484(81.57%)	23167(80.44%)		23456(72.02%)	17599(71.34%)	
Family history	No	23934(73.72%)	20931(72.67%)	0.004	24167(74.20%)	18177(73.68%)	0.163
	Yes	8532(26.28%)	7871(27.33%)		8403(25.80%)	6493(26.32%)	
Oral contraception	No				14751(45.29%)	11057(44.82%)	0.266
	Yes				17819(54.71%)	13613(55.18%)	
HRT	No				10727(32.94%)	7747(31.40%)	<0.001
	Yes				21843(67.06%)	16923(68.60%)	

BMI, body mass index. HRT, hormone replace therapy.

### Effectiveness of joint cancer screening on primary endpoints

After a median follow-up of 10.48 years for incidence and 16.85 years for mortality, a total of 5,506 PLCO cancer cases (4,636 males and 870 females), 1,845 PLCO cancer deaths (1,227 males and 618 females), 3,970 all-neoplasms deaths (2,576 males and 1,394 females), and 14,221 all-cause deaths (9,461 males and 4,760 females) were documented in the screening arm, while the numbers in the control arm were 6,261 (5,054 males and 1,207 females), 2,417 (1,531 males and 886 females), 5,091 (3,109 males and 1,982 females), and 18,516 (11,514 males and 7,002 females) in the control arm, respectively (**Table 2**).

**Table 2:** Gender-specific effects of joint cancer screening on primary endpoints by risk groups.

Outcomes	Control arm			Screening arm			Adjusted HR (95%CI)	P value
	N	Events	Event Rate/10000 PYs	N	Events	Event Rate/10000 PYs		
Male^*^								
PLCO cancers incidence								
Low risk	12998	1568	113.81(108.27,119.54)	11926	1525	119.20(113.31,125.28)	1.04(0.97,1.12)	0.251
High risk	19468	3486	173.57(167.88,179.40)	16876	3111	176.98(170.83,183.27)	1.03(0.98,1.08)	0.309
Overall	32466	5054	149.26(145.18,153.41)	28802	4636	152.64(148.29,157.08)	1.03(0.99,1.08)	0.131
PLCO cancers mortality								
Low risk	12998	327	15.54(13.91,17.28)	11926	274	13.83(12.26,15.53)	0.87(0.74,1.03)	0.097
High risk	19468	1204	40.85(38.59,43.20)	16876	953	36.18(33.93,38.53)	0.88(0.81,0.96)	**0.004**
Overall	32466	1531	30.30(28.81,31.85)	28802	1227	26.58(25.12,28.10)	0.88(0.82,0.95)	**0.001**
All-neoplasms mortality								
Low risk	12998	792	37.63(35.07,40.31)	11926	684	34.52(31.99,37.17)	0.90(0.81,0.99)	**0.048**
High risk	19468	2317	78.61(75.45,81.86)	16876	1892	71.83(68.64,75.11)	0.91(0.86,0.97)	**0.002**
Overall	32466	3109	61.54(59.40,63.73)	28802	2576	55.81(53.68,57.99)	0.91(0.86,0.96)	**0.003**
All-cause mortality								
Low risk	12998	2669	126.82(122.07,131.69)	11926	2208	111.42(106.83,116.13)	0.86(0.82,0.92)	**<0.001**
High risk	19468	8845	300.09(293.88,306.39)	16876	7255	275.43(269.14,281.82)	0.91(0.89,0.94)	**<0.001**
Overall	32466	11514	227.91(223.77,232.10)	28802	9461	204.97(200.87,209.13)	0.90(0.88,0.93)	**<0.001**
Female^¶^								
PLCO cancers incidence								
Low risk	24478	584	21.57(19.87,23.37)	18928	463	21.99(20.04,24.05)	1.03(0.91,1.16)	0.657
High risk	8092	623	72.65(67.09,78.51)	5742	407	65.60(59.43,72.18)	0.91(0.80,1.03)	0.125
Overall	32570	1207	33.86(31.99,35.81)	24670	870	31.91(29.84,34.08)	0.96(0.88,1.05)	0.391
PLCO cancers mortality								
Low risk	24478	368	9.13(8.23,10.10)	18928	272	8.58(7.59,9.63)	0.94(0.80,1.10)	0.445
High risk	8092	518	42.19(38.67,45.94)	5742	346	38.23(34.34,42.40)	0.90(0.79,1.03)	0.132
Overall	32570	886	16.86(15.77,17.99)	24670	618	15.16(13.99,16.39)	0.91(0.82,1.01)	0.083
All-neoplasms mortality								
Low risk	24478	1110	27.55(25.96,29.21)	18928	802	25.29(23.58,27.08)	0.92(0.84,1.01)	0.070
High risk	8092	872	71.04(66.42,75.85)	5742	592	65.42(60.29,70.83)	0.91(0.82,1.01)	0.079
Overall	32570	1982	37.71(36.07,39.39)	24670	1394	34.20(32.43,36.02)	0.91(0.85,0.98)	**0.009**
All-cause mortality								
Low risk	24478	3896	96.71(93.71,99.78)	18928	2754	86.84(83.64,90.13)	0.91(0.87,0.95)	**<0.001**
High risk	8092	3106	253.02(244.23,262.02)	5742	2006	221.66(212.10,231.50)	0.86(0.81,0.91)	**<0.001**
Overall	32570	7002	133.22(130.12,136.36)	24670	4760	116.77(113.49,120.12)	0.88(0.85,0.92)	**<0.001**

PYs, person years. *, adjusting age, smoking, diabetes, prostate surgery, family history. ¶, adjusting age, smoking, diabetes, ovary surgery, organ-related disease, oral contraception and family history. The bold values in **Table 2** indicate P values of less than 0.05.

**Figure 1:**
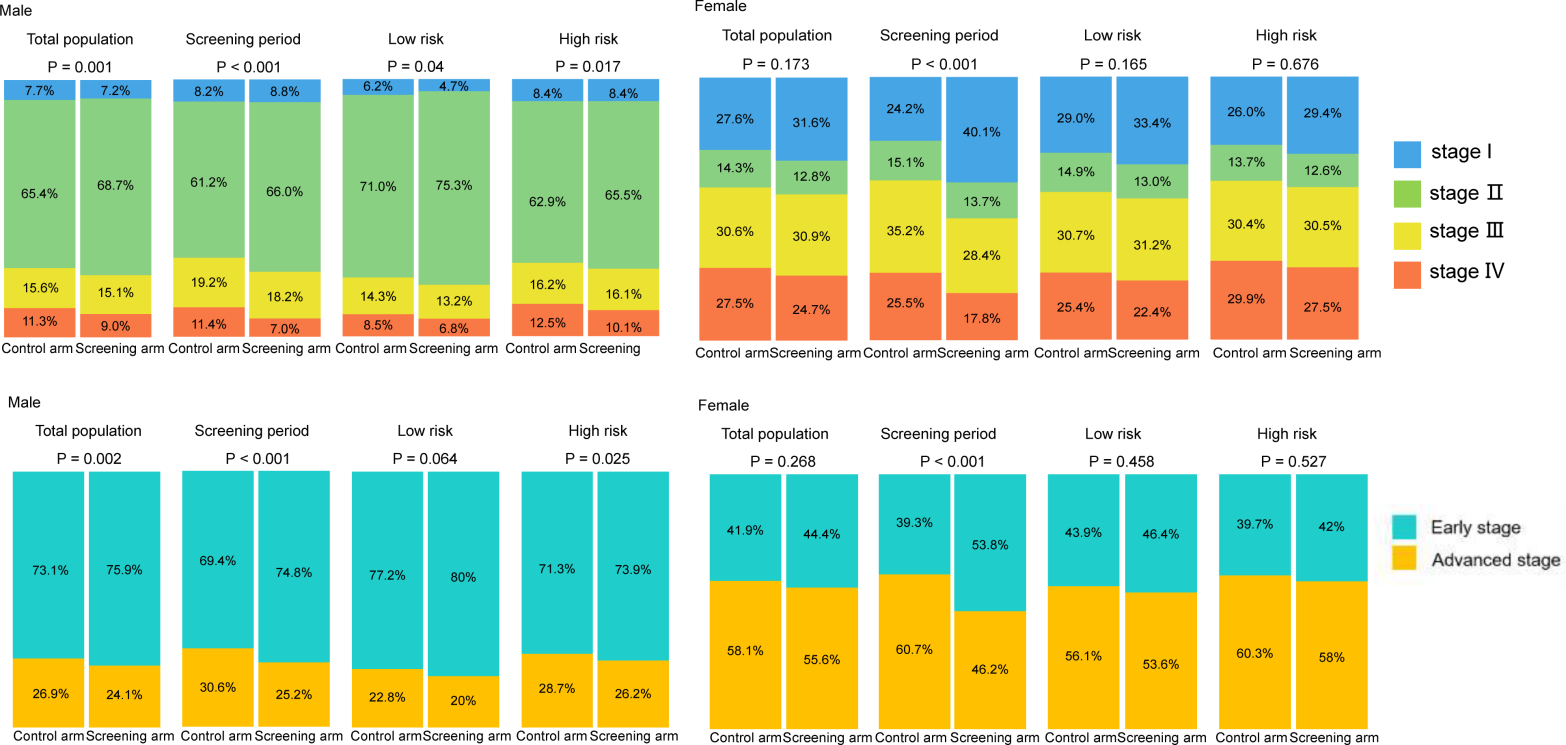
Gender-specific effects of joint cancer screening on cancer stage shift. Missing data of cancer stage were not included in this figure.

**Figure 2:**
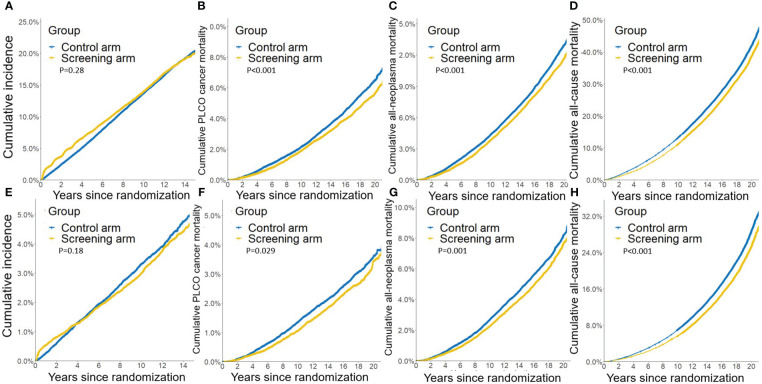
Gender-specific effects of joint cancer screening on crude PLCO cancers incidence **(A, E)**, PLCO cancers mortality **(B, F)**, all neoplasms mortality **(C, G)**, and all-cause mortality **(D, H)**. **(A, B, C, D)** for males, and **(E, F, G, H)** for females.

Compared to the control arm, joint cancer screening significantly increased the percent of male early-stage (stage I and II) PLCO cancers across the whole study period (75.9% [3,423/4,511] vs. 73.1% [3,594/4,914], P value = 0.002) and the screening period (74.8% [1,363/1,822] vs. 69.4% [1,086/1,565], P value < 0.001), while joint cancer screening only significantly increased female early-stage PLCO cancers in the screening period (53.8% [157/292] vs. 39.3% [151/384], P value < 0.001) (**Figure 1**). Moreover, as observed in **Figure 2**, in both males and females, log-rank tests showed a non-significant difference in PLCO cancer incidence between the screening arm and control arm (P values of 0.28 for males and 0.18 for females), but significantly lower PLCO cancer mortality (P values <0.001 for males and 0.029 for females), all-neoplasms mortality (P values <0.001 for males and 0.001 for females) and all-cause mortality (P values for both males and females <0.001) for the screening arm compared to the control arm.

As shown in **Supplementary Tables 1**, **2**, age, smoking, diabetes, prostate surgery, family history were included into the male PLCO-CA model, while age, smoking, diabetes, ovarian surgery, organ-related disease, oral contraception and family history were included into the female PLCO-CA model. After adjusting all available factors associated with gender-specific PLCO cancer **(Supplementary Tables 1**, **2**), joint cancer screening did not significantly reduce gender-specific PLCO cancer incidence, with HR (95%CIs) of 1.03(0.99, 1.08) for male (P value = 0.131) and 0.96(0.88, 1.05) for female (P value = 0.391). However, joint cancer screening significantly reduced male-specific PLCO cancer mortality [HR (95%CIs): 0.88(0.82, 0.95), P value = 0.001]. More importantly, joint cancer screening significantly reduced both gender-specific all-neoplasm mortality [HR (95%CIs) of 0.91(0.86, 0.96) for males (P value = 0.003) and 0.91(0.85, 0.98) for female (P value = 0.009)] and all-cause mortality [HR (95%CIs) of 0.90(0.88, 0.93) for male (P value <0.001) and 0.88(0.85, 0.92) for female (P value <0.001)] (**Table 2**).

As showed in **Supplementary Figure 2**, after excluding CRC-associated outcomes, further CRC-free sensitivity analyses showed that joint cancer screening significantly increased the CRC-free PLCO cancer incidence [HR (95%CIs): 1.07(1.03, 1.11), P value = 0.008], but did not significantly reduce the CRC-free PLCO cancer mortality [HR (95%CIs): 0.98(0.91, 1.04), P value = 0.485]. However, joint cancer screening significantly reduced both CRC-free all-neoplasm mortality [HR (95%CIs) of 0.94(0.90, 0.98)] and CRC-free all-cause mortality [HR (95%CIs) of 0.91(0.88, 0.93), P value <0.001].

### Subgroup analyses on the effectiveness of risk-stratified joint cancer screening

As shown in **Supplementary Figure 3**, gender-specific PLCO-CA models showed good discrimination (AUCs of 0.60 (0.47, 0.64) for males and 0.71 (0.65, 0.73) for females) and good calibration(O/E ratios of 0.99(0.97, 1.03) for males and 1.00(0.93, 1.07) for females, both P values >0.05) in the derivation cohort. Similar discrimination (AUCs of 0.59(0.56, 0.61) for males and 0.70 (0.65, 0.73) for females, both P values for differences between gender-specific derivation and validation AUCs >0.05) and calibration(both P values for O/E ratios >0.05 in males and females) were observed in the validation cohort. After reclassifying the participants into low- and high-risk subgroups according to the optimal cutoff value of ROCs for 10-year gender-specific PLCO-CA prediction risk. We observed similar gender-specific effectiveness of joint cancer screening in both low- and high-risk subgroups as the preliminary analyses (**Table 2**; **Supplementary Figures 2**, **4**, **5**).

### Meta-analyses of gender-specific effectiveness of joint cancer screening

**Figure 3:**
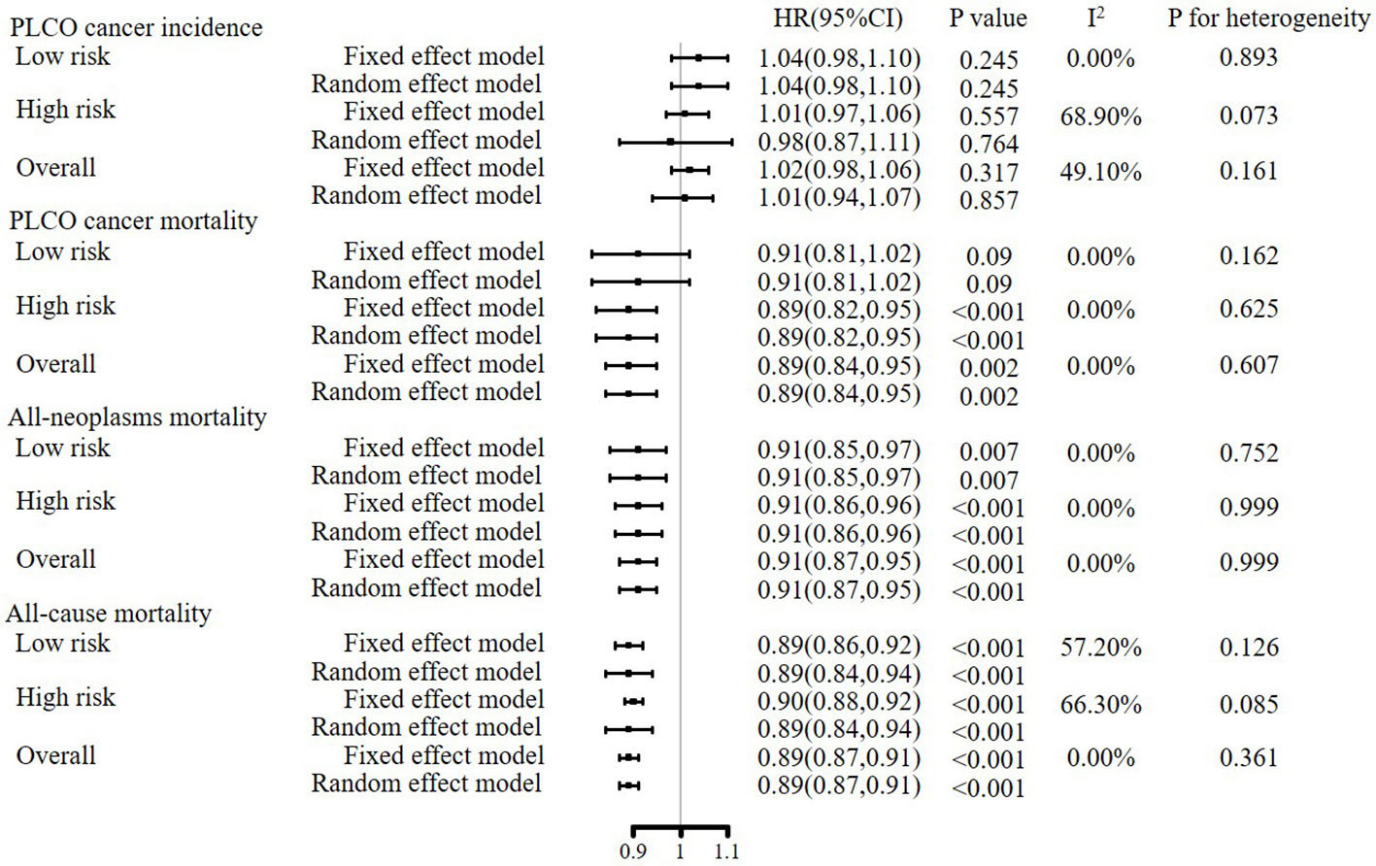
Meta-analyses of gender-specific effects of joint cancer screening on primary endpoints.

As shown in **Figure 3**, in both fixed and random effect models, meta-analyses of gender-specific effectiveness of joint cancer screening showed that joint cancer screening did not significantly reduce pooled PLCO cancer incidence [HR (95%CIs) for random effect model: 1.01 (0.94, 1.07), P value = 0.845], but significantly reduced pooled PLCO cancer mortality [HR (95%CIs) for random effect model: 0.89(0.84, 0.95), P value = 0.002], all-neoplasms mortality [HR (95%CIs) for random effect model: 0.91(0.87, 0.95), P value < 0.001] and all-cause mortality[HR (95%CIs) for random effect model: 0.89(0.87, 0.91), P value < 0.001]. Meta-analyses of subgroup analyses showed almost the same results as the primary meta-analyses (**Figure 3**).

### Sensitivity analysis on the effectiveness of joint cancer screening by compliance

**Figure 4:**
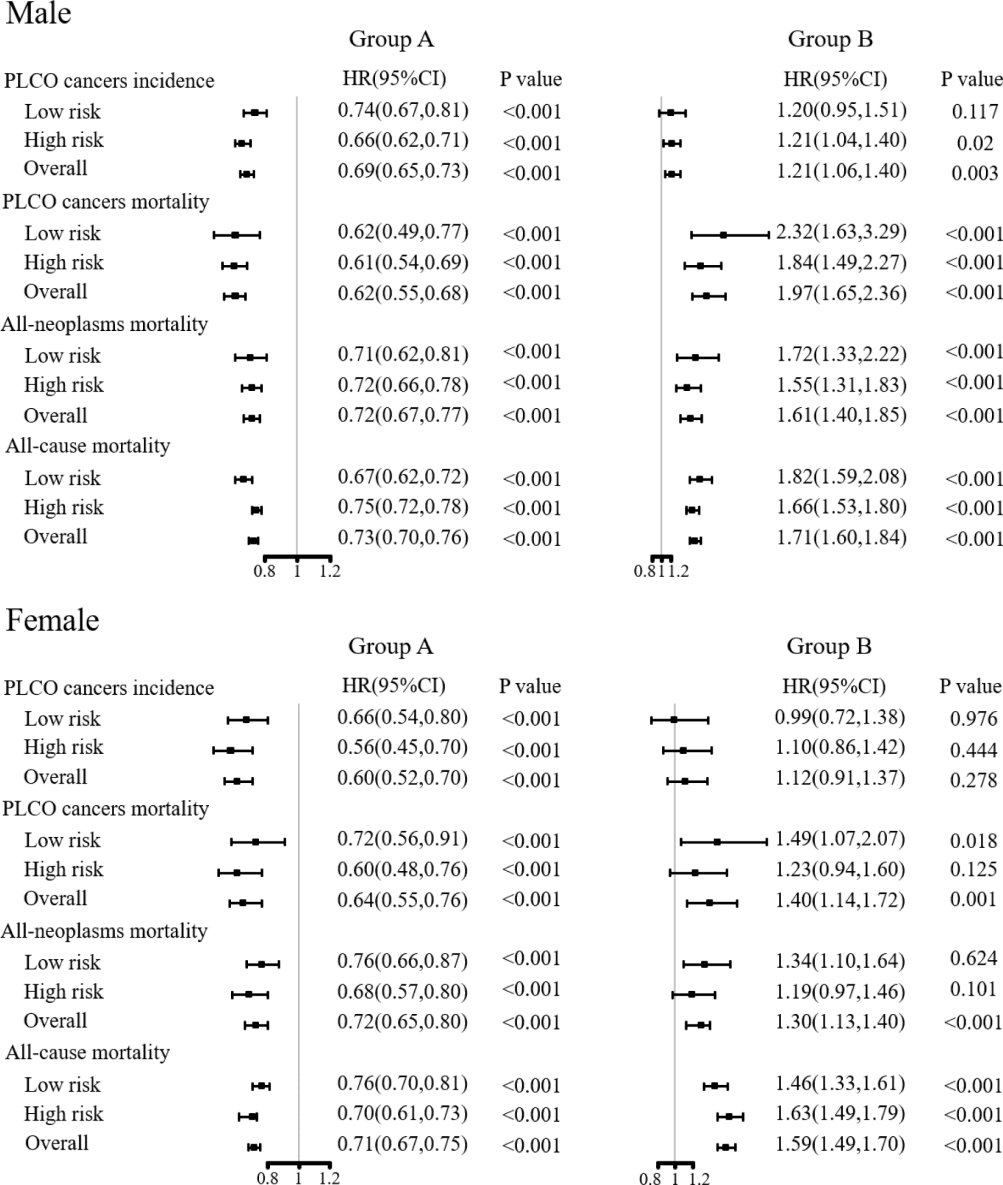
Sensitivity analyses on the effects of joint cancer screening by compliance. Group A was defined as complete compliance with the screening protocol. Group B was defined as never receiving any screening examinations or tests in the screening arm.

As shown in **Figure 4**; **Supplementary Figure 6**, compared to the control arm, participants who complied with the screening protocol and received all rounds of joint cancer screening had significantly decreased PLCO cancer incidence [HR (95%CIs) of 0.69(0.65, 0.73) for males and 0.60(0.52, 0.70) for female], PLCO cancer mortality [HR (95%CIs) of 0.62(0.55, 0.68) for male and 0.65(0.55, 0.76) for female], all-neoplasms mortality [HR (95%CIs) of 0.72(0.67, 0.77) for male and 0.72(0.65, 0.80) for female] and all-cause mortality [HR (95%CIs) of 0.73(0.70, 0.76) for male and 0.71(0.67, 0.75) for female]. On contrary, participants who were assigned to the screening arm but did not receive any exam had significantly increased PLCO cancer incidence [HR (95%CIs) of 1.21(1.06, 1.40)] for male, both gender-specific increased PLCO cancer mortality [HR (95%CIs) of 1.97(1.65, 2.36) for male and 1.40(1.41, 1.72) for female], all-neoplasms mortality [HR (95%CIs) of 1.61(1.40, 1.85) for male and 1.30(1.13, 1.40) for female] and all-cause mortality [HR (95%CIs) of 1.71(1.60, 1.84) for male and 1.59(1.49, 1.70) for female]. Subgroup analyses showed similar results as observed in the preliminary subgroup analyses.

## Discussion

In this study based on PLCO cancer screening trial, joint cancer screening means that individuals in the screening group received multiple methods recommended by the guidelines to screen for different cancers during the same period. To our knowledge, based on the PLCO trial, this is the first study to investigate the effectiveness of joint cancer screening, and this is also the first intervention study to explore the potential benefits of risk-stratified joint cancer screening based on easy-to-use combined cancer risk assessment. According to previous results of the PLCO trial (9, 27–29), compared with the control group, four individual screenings on PLCO cancer-specific incidence were non-significant 5% increase for lung cancer, significant 22% increase for prostate cancer, non-significant 21% increase for ovarian cancer and significant 21% decrease for colorectum cancer, while effects on cancer-specific mortality were non-significant 1% increase for lung cancer, non-significant 13% increase for prostate cancer, non-significant 18% increase for ovarian cancer and significant 26% increase for colorectum cancer. If there were no synergies and spillover effects between individual screenings, the effect sum of four individual screenings should be 27% increase for PLCO cancer incidence and 4% increase for PLCO cancer mortality, respectively. However, in this study, the pooled effects of joint screening compared to the control were non-significant 1% increase for cancer incidence and significant 11% decrease for cancer mortality, respectively. Both of them in this study were better than the simple effect sum of four individual screenings. For those with better adherence to joint screening, more benefits can be observed, with significant 38% and 36% decrease in cancer mortality for males and females, respectively. The joint decrease in cancer mortality would further climbed to 39% and 40% for high-risk males and females with better adherence. All of these might suggest a potential synergy between the individual screenings. Therefore, joint cancer screening should be recommended as a potentially more suitable choice to reduce the overall cancer burden than single cancer screening. Moreover, researches are needed in the future to explore and confirm the synergistic effect of joint cancer screening.

In this study, PLCO joint cancer screening significantly reduce PLCO cancer mortality but did not reduce incidence. Two major reasons would lead to the non-significant reduction of PLCO cancer incidence after joint screening. First, only colorectal cancer had well-defined precancerous lesions, and other three PLCO cancers (prostate, lung, and ovarian cancer) had no precancerous lesions. Secondly, previous studies from the PLCO trial demonstrated that only FSG screening could effectively lead to early-stage shift of CRC (9), but no significant early-stage shift was observed for PSA combined with DRE screening for prostate cancer (28), CA125 combined with TVU screening for ovarian cancer (29), and chest radiograph screening for lung cancer (27). Additionally, non-adherence to screening protocol, contamination in the control arm, potential overdiagnosis associated with screening, and possible dilution of screening effect after long-term follow-up, might collectively result in non-significant reduction in PLCO cancer incidence (9, 27–29). Furthermore, joint screening significantly reduced male-specific PLCO cancer mortality, but not among females. Several reasons would lead to the gender-specific joint cancer screening effects. First, although men have a higher cancer incidence rate than women due to gender-specific hormonal, behavioral and genetic differences, female patients often present with higher-grade disease and experience worse outcomes (1, 33–37). Secondly, CRC screening has been observed to be less effective in women than in men (9, 38), and lung cancer screening often demonstrates equal benefits between males and females (39). More studies are needed to validate the gender-specific joint cancer screening effects.

Joint cancer screening could not overcome the above limitations within a single cancer screening. On contrary, joint cancer screening may magnify these potential limitations into a relatively more obvious limitation. For example, the “all-versus-none” design of joint cancer screening would significantly increase the difficulty of implementing integrated interventions in the screening arm, which would likely to weaken the adherence to any single intervention. Participants in the control arm may also significantly increase the intentions of active seeking for any or all screening components within the joint cancer screening due to multiple informed consents, thereby increasing the likelihood of contamination in the control arm. Multiple false-positive results from joint cancer screening would likely to lead to more over-examination and over-diagnosis compared to a single false-positive result in the screening arm. Additionally, all participants in the joint cancer screening had to meet more indications for multiple screening exams compared to single cancer screening, hence increasing the bias of healthy volunteers. All these amplified limitations would collectively dilute the expected effectiveness of single cancer screening.

Even with the above-mentioned potentially amplified limitations, our analyses still showed a significant reduction in PLCO cancer mortality, all-neoplasms mortality, and all-cause mortality associated with joint cancer screening. Further CRC-free sensitivity analyses showed that joint cancer screening significantly increased the CRC-free PLCO cancer incidence and did not reduce the CRC-free PLCO cancer mortality, which were similar to the previous single cancer screening (27–29). However, more importantly, joint cancer screening still significantly reduced both CRC-free all-neoplasm mortality and all-cause mortality. These results suggested that there must be some important difference between joint and single cancer screening to offset and even overweigh limitations mentioned above. Although both joint cancer screening and four individual cancer screening use the similar methods recommended by the guidelines, combination of these methods scheduled at the same period would bring an additional health promotion effects compared with any single method scheduled in different periods due to the cumulative health concerns. When participants received any positive result in either joint or single cancer screening, their physicians would not only discuss with the participants to determine which diagnostic evaluation procedures would be performed, but also probably provide several health promotion suggestions on cancer prevention, such as quitting smoking, alcohol abstinence, exercising, weight control, adopting a healthy diet, increasing preventive medication (such as NSAIDs), and so on. Unlike a positive result in single cancer screening, multiple positive results from joint cancer screening would probably enhance more adoption of several health promotion behaviors due to the multiple suggestions by different physicians during the same period. The enhanced adoption of health promotion behaviors will not only prevent target cancers, but also prevent other cancers and chronic diseases (such as cardiovascular diseases) with shared risk factors (40–44). That’s would be most likely reason why joint cancer screening could reduce the risks of all-neoplasm mortality and all-cause mortality. Additionally, multiple positive screening results would also strengthen the adherence to regular screening behavior due to the same cumulative health concerns. This may also partly explain why participants who completely followed multiple rounds of joint screening received more benefits in PLCO cancer mortality, all-neoplasms mortality, and all-cause mortality than those without good adherence to the multiple screening.

In addition to the health promotion difference between joint and single cancer screening modes, another two differences also deserved attentions. First, cost difference. This difference did not lie in the screening cost but in the logistic costs. Joint screening only requires one time of population recruitment, screening organization, information collection, etc., while single cancer screening requires independent organization for different cancers, thereby increasing the costs of screening organization. Second, effectiveness difference. For example, in a single lung cancer screening practice, residents who participate in screening may not end up dying from lung cancer, but may die from colorectal cancer, as the investigators primarily focus on lung cancer-related outcomes. The same risk would be observed in other single cancer screening. However, this risk would be significantly reduced in joint screening for lung and colorectal cancer. Although the frequency of screening varies for different cancers, most cancers are screened at intervals of 1, 2, 3 or 5 years. Therefore, the joint screening of different cancers can be well arranged in different intervals. In summary, due to the enhanced adoption of health promotion behaviors, potential reduced overall cost, and increased effectiveness, joint cancer screening scheduled at different cancer-specific intervals during the same period would be recommended to improve the overall cost-effectiveness of current single cancer screening and expected to reduce the overall cancer burden in the future.

Moreover, we developed gender-specific PLCO-CA models based on modifiable risk factors to predict the combined risk of prostate, lung, colorectal, and ovarian cancer. Although several guidelines have detailed cancer screening recommendation according to the age (13, 14, 45–47), this age-stratified screening recommendation could not effectively identify persons with other risk factors of cancer. Currently, several studies had suggested that risk-stratified screening strategy integrating multiple factors (both genetic and non-genetic factors) would be more effective in identifying people at high risk of cancer and improving the potential screening effect (48–51). Moreover, consistent with previous studies (52), significant reduction of PLCO cancer mortality was only observed in participants at high risk of multiple cancer but not in those at low risk. All of these suggested that integrated risk-stratified screening scheme would be more important in joint cancer screening. However, as mentioned above, compared with single cancer screening, joint cancer screening not only requires more resources and manpower but also is relatively difficult to organize. Therefore, in resource-limited settings, we still recommend joint cancer screening for participants at high risk of multiple cancers or pan-cancer. In addition, although the AUCs of the gender-specific PLCO-CA models in this study were relatively good, there is still much room to improve the overall prediction discrimination. Further studies have confirmed that integrating polygenic risk scores with modifiable risk factors improves risk prediction of pan-cancer (53). Moreover, liquid biopsy markers, such as methylation signatures in cell-free DNA, are expected to have a huge potential value in early detection of pan-cancer (54–56).

Last but not the least, as in the PLCO trial, the joint cancer screening has been initiated in some developed countries. For example, a 55-year-old female smoker in the United States will be regularly screened for breast cancer (mammogram), cervical cancer (HPV test and/or a pap test), colorectal cancer (colonoscopy or stool-based test), and lung cancer (low-dose CT scan), which are initiated by her primary care physician. However, primary care in most other countries does not cover all these screening tests for above cancers. Therefore, integrating the scattered single cancer screening resources into a unified joint screening model would be a relatively optimal choice advocated in countries where joint screening is not yet available. Second, in addition to the cancers jointly screened in the United States, it is important to designate a country-specific joint cancer screening program based on their cancer burden priority. Third, joint cancer screening is not just the combination of conventional tests screening for different cancers separately, it could also be a unified method, such as liquid biopsy, to screen for different cancers. Fourth, since the synergistic effect of joint screening would probably attributable to non-cancer specific effects, the effects of cancer-specific screening between joint screening and single screening are probably not the same. Future RCTs with parallel arms are needed to investigate this difference and further support the synergistic effect of joint screening.

In addition to the several strengths above, there are also several limitations in this study. First, as the PLCO trial was the only published RCT for joint cancer screening in the world until now, there is currently no suitable study for external validation. Although both subgroup analyses and sensitivity analyses yielded very similar effects of joint cancer screening, the current results are based on one RCT study with specific population groups and specific age group. Therefore, it could be expected the current findings would not be broadly applicable to other population groups. Second, changes in health behavior after multiple positive screenings may be an important factor associated with all-neoplasms mortality and all-cause mortality, especially adoption of healthier lifestyle after positive cancer screens. Future studies with more sophisticated designs and more exploration are needed to further support this hypothesis. Third, joint cancer screening is expected to be more cost-effective than single cancer screening. However, the PLCO trial did not collect the data needed for health economic evaluation. Additionally, the long latency period, high disease prevalence, and significant associated morbidity and mortality are the three basic criteria for risk-reduction cancer screening and intervention. Latency period significantly varies across different cancers. According to a recent study, the estimated median latency period of lung, breast, ovarian, and rectum cancers was 13.6, 16.3, 44.1, and 29.8 years, respectively (57). Moreover, the latency period of cancer would be modulated by different risk factors, such as smoking, ionizing radiation exposure, special occupation exposure, and use of cancer-prevention medicine (58–60). Although a median follow-up of 10.48 years for incidence and 16.85 years for mortality were recorded in this study, it would not be long enough to capture meaningful outcomes.

## Conclusion

Based on the unique “all-versus-none” RCT design, joint cancer screening can significantly reduce PLCO cancer mortality, all-neoplasms mortality, and all-cause mortality. More compliance, more benefits. Therefore, joint cancer screening could be recommended as a potentially more suitable choice to reduce the overall cancer burden than traditional single cancer screening. However, notably, organizing a joint cancer screening not only requires more resources, manpower and ingenious design, but also needs more attentions to the potential harms. Although the current results support that joint cancer screening can also benefits the low-risk populations, we still prioritize joint cancer screening for high-risk groups to reduce the difficulty of joint intervention and improve the potential cost-effectiveness.

## Data availability statement

The original contributions presented in the study are included in the article/**Supplementary Material**. Further inquiries can be directed to the corresponding authors.

## Ethics statement

Written informed consent was obtained from the individual(s) for the publication of any potentially identifiable images or data included in this article.

## Author contributions

ZF: Writing – original draft. YZ: Writing – original draft. QY: Writing – review & editing, Data curation, Methodology, Formal analysis, Software. XL: Data curation, Writing – review & editing. HD: Formal Analysis, Writing – review & editing. YL: Formal Analysis, Writing – review & editing. SC: Data curation, Writing – review & editing. ZL: Writing – review & editing. LY: Writing – review & editing. FaS: Writing – review & editing. YH: Writing – review & editing. FeS: Writing – review & editing.

## References

[1] Sung H Ferlay J Siegel RL Laversanne M Soerjomataram I Jemal A. Global cancer statistics 2020: GLOBOCAN estimates of incidence and mortality worldwide for 36 cancers in 185 countries. CA Cancer J Clin. (2021) 71:209–49. doi: 10.3322/caac.21660 33538338

[2] Ahmad FB Anderson RN. The leading causes of death in the US for 2020. JAMA. (2021) 325:1829–30. doi: 10.1001/jama.2021.5469 PMC8145781 33787821

[3] Diseases GBD Injuries C. Global burden of 369 diseases and injuries in 204 countries and territories, 1990-2019: a systematic analysis for the Global Burden of Disease Study 2019. Lancet. (2020) 396:1204–22. doi: 10.1016/S0140-6736(20)30925-9 PMC7567026 33069326

[4] Qiu H Cao S Xu R. Cancer incidence, mortality, and burden in China: a time-trend analysis and comparison with the United States and United Kingdom based on the global epidemiological data released in 2020. Cancer Commun (Lond). (2021) 41:1037–48. doi: 10.1002/cac2.12197 PMC8504144 34288593

[5] Siegel RL Miller KD Fuchs HE Jemal A. Cancer statistics, 2022. CA Cancer J Clin. (2022) 72:7–33. doi: 10.3322/caac.21708 35020204

[6] Independent UKPoBCS. The benefits and harms of breast cancer screening: an independent review. Lancet. (2012) 380:1778–86. doi: 10.1016/S0140-6736(12)61611-0 23117178

[7] National Lung Screening Trial Research T Aberle DR Adams AM Berg CD Black WC Clapp JD. Reduced lung-cancer mortality with low-dose computed tomographic screening. N Engl J Med. (2011) 365:395–409. doi: 10.1056/NEJMoa1102873 21714641 PMC4356534

[8] de Koning HJ van der Aalst CM de Jong PA Scholten ET Nackaerts K Heuvelmans MA. Reduced lung-cancer mortality with volume CT screening in a randomized trial. N Engl J Med. (2020) 382:503–13. doi: 10.1056/NEJMoa1911793 31995683

[9] Schoen RE Pinsky PF Weissfeld JL Yokochi LA Church T Laiyemo AO. Colorectal-cancer incidence and mortality with screening flexible sigmoidoscopy. N Engl J Med. (2012) 366:2345–57. doi: 10.1056/NEJMoa1114635 PMC3641846 22612596

[10] Nishihara R Wu K Lochhead P Morikawa T Liao X Qian ZR. Long-term colorectal-cancer incidence and mortality after lower endoscopy. N Engl J Med. (2013) 369:1095–105. doi: 10.1056/NEJMoa1301969 PMC3840160 24047059

[11] Sankaranarayanan R Nene BM Shastri SS Jayant K Muwonge R Budukh AM. HPV screening for cervical cancer in rural India. N Engl J Med. (2009) 360:1385–94. doi: 10.1056/NEJMoa0808516 19339719

[12] Hugosson J Roobol MJ Mansson M Tammela TLJ Zappa M Nelen V. A 16-yr follow-up of the european randomized study of screening for prostate cancer. Eur Urol. (2019) 76:43–51. doi: 10.1016/j.eururo.2019.02.009 30824296 PMC7513694

[13] Force USPST Krist AH Davidson KW Mangione CM Barry MJ Cabana M. Screening for lung cancer: US preventive services task force recommendation statement. JAMA. (2021) 325:962–70. doi: 10.1001/jama.2021.1117 33687470

[14] Force USPST Grossman DC Curry SJ Owens DK Bibbins-Domingo K Caughey AB. Screening for prostate cancer: US preventive services task force recommendation statement. JAMA. (2018) 319:1901–13. doi: 10.1001/jama.2018.3710 29801017

[15] Myers ER Moorman P Gierisch JM Havrilesky LJ Grimm LJ Ghate S. Benefits and harms of breast cancer screening: A systematic review. JAMA. (2015) 314:1615–34. doi: 10.1001/jama.2015.13183 26501537

[16] Lin JS Perdue LA Henrikson NB Bean SI Blasi PR. Screening for colorectal cancer: updated evidence report and systematic review for the US preventive services task force. JAMA. (2021) 325:1978–98. doi: 10.1001/jama.2021.4417 PMC13509038 34003220

[17] Force USPST Curry SJ Krist AH Owens DK Barry MJ Caughey AB. Screening for cervical cancer: US preventive services task force recommendation statement. JAMA. (2018) 320:674–86. doi: 10.1001/jama.2018.10897 30140884

[18] Li N Tan F Chen W Dai M Wang F Shen S. One-off low-dose CT for lung cancer screening in China: a multicenter, population-based, prospective cohort study. Lancet Respir Med. (2022) 10:378–91. doi: 10.1016/S2213-2600(21)00560-9 35276087

[19] Chen R Liu Y Song G Li B Zhao D Hua Z. Effectiveness of one-time endoscopic screening program in prevention of upper gastrointestinal cancer in China: a multicenter population-based cohort study. Gut. (2021) 70:251–60. doi: 10.1136/gutjnl-2019-320200 PMC7815635 32241902

[20] Wei WQ Chen ZF He YT Feng H Hou J Lin DM. Long-term follow-up of a community assignment, one-time endoscopic screening study of esophageal cancer in China. J Clin Oncol. (2015) 33:1951–7. doi: 10.1200/JCO.2014.58.0423 PMC4881309 25940715

[21] Menon U Gentry-Maharaj A Burnell M Singh N Ryan A Karpinskyj C. Ovarian cancer population screening and mortality after long-term follow-up in the UK Collaborative Trial of Ovarian Cancer Screening (UKCTOCS): a randomized controlled trial. Lancet. (2021) 397:2182–93. doi: 10.1016/S0140-6736(21)00731-5 PMC8192829 33991479

[22] Su F Weiss NS Beste LA Moon AM Jin GY Green P. Screening is associated with a lower risk of hepatocellular carcinoma-related mortality in patients with chronic hepatitis B. J Hepatol. (2021) 74:850–9. doi: 10.1016/j.jhep.2020.11.023 PMC8045451 33245934

[23] Huang Y Wang H Lyu Z Dai H Liu P Zhu Y. Development and evaluation of the screening performance of a low-cost high-risk screening strategy for breast cancer. Cancer Biol Med. (2021) 19:1375–84. doi: 10.20892/j.issn.2095-3941.2020.0758 PMC9500221 34570443

[24] Liu X Zhang Y Duan H Yang L Sheng C Fan Z. Risk-stratified multi-round PSA screening for prostate cancer integrating the screening reference level and subgroup-specific progression indicators. Eur J Med Res. (2023) 28:257. doi: 10.1186/s40001-023-01228-x 37496058 PMC10369696

[25] Huang Y Lyu Z Zhang Y Liu X Zhang Y Liu Y. Cohort profile: design and methods of the Chinese colorectal, breast, lung, liver, and stomach cancer screening trial (C-BLAST). Cancer Biol Med. (2023) 20:713–20. doi: 10.20892/j.issn.2095-3941.2023.0278 PMC10618950 37905555

[26] Gohagan JK Prorok PC Greenwald P Kramer BS. The PLCO cancer screening trial: Background, goals, organization, operations, results. Rev Recent Clin Trials. (2015) 10:173–80. doi: 10.2174/1574887110666150730123004 26238115

[27] Oken MM Hocking WG Kvale PA Andriole GL Buys SS Church TR. Screening by chest radiograph and lung cancer mortality: the Prostate, Lung, Colorectal, and Ovarian (PLCO) randomized trial. JAMA. (2011) 306:1865–73. doi: 10.1001/jama.2011.1591 22031728

[28] Andriole GL Crawford ED Grubb RL 3rd Buys SS Chia D Church TR. Mortality results from a randomized prostate-cancer screening trial. N Engl J Med. (2009) 360:1310–9. doi: 10.1056/NEJMoa0810696 PMC2944770 19297565

[29] Buys SS Partridge E Black A Johnson CC Lamerato L Isaacs C. Effect of screening on ovarian cancer mortality: the Prostate, Lung, Colorectal and Ovarian (PLCO) Cancer Screening Randomized Controlled Trial. JAMA. (2011) 305:2295–303. doi: 10.1001/jama.2011.766 21642681

[30] Prorok PC Andriole GL Bresalier RS Buys SS Chia D Crawford ED. Design of the prostate, lung, colorectal and ovarian (PLCO) cancer screening trial. Control Clin Trials. (2000) 21:273S–309S. doi: 10.1016/S0197-2456(00)00098-2 11189684

[31] Higgins JP Thompson SG Deeks JJ Altman DG. Measuring inconsistency in meta-analyses. BMJ. (2003) 327:557–60. doi: 10.1136/bmj.327.7414.557 PMC192859 12958120

[32] Tsilidis KK Kasimis JC Lopez DS Ntzani EE Ioannidis JP. Type 2 diabetes and cancer: umbrella review of meta-analyses of observational studies. BMJ. (2015) 350:g7607. doi: 10.1136/bmj.g7607 25555821

[33] Mederos N Friedlaender A Peters S Addeo A. Gender-specific aspects of epidemiology, molecular genetics and outcome: lung cancer. ESMO Open. (2020) 5:e000796. doi: 10.1136/esmoopen-2020-000796 33148544 PMC7643520

[34] Doshi B Athans SR Woloszynska A. Biological differences underlying sex and gender disparities in bladder cancer: current synopsis and future directions. Oncogenesis. (2023) 12:44. doi: 10.1038/s41389-023-00489-9 37666817 PMC10477245

[35] Zhu Y Shao X Wang X Liu L Liang H. Sex disparities in cancer. Cancer Lett. (2019) 466:35–8. doi: 10.1016/j.canlet.2019.08.017 31541696

[36] Lopes-Ramos CM Quackenbush J DeMeo DL. Genome-wide sex and gender differences in cancer. Front Oncol. (2020) 10:597788. doi: 10.3389/fonc.2020.597788 33330090 PMC7719817

[37] Dorak MT Karpuzoglu E. Gender differences in cancer susceptibility: an inadequately addressed issue. Front Genet. (2012) 3:268. doi: 10.3389/fgene.2012.00268 23226157 PMC3508426

[38] Holme Ø Løberg M Kalager M Bretthauer M Hernán MA Aas E. Long-term effectiveness of sigmoidoscopy screening on colorectal cancer incidence and mortality in women and men: A randomized trial. Ann Intern Med. (2018) 168:775–82. doi: 10.7326/M17-1441 PMC6853067 29710125

[39] Randhawa S Sferra SR Das C Kaiser LR Ma GX Erkmen CP. Examining gender differences in lung cancer screening. J Community Health. (2020) 45:1038–42. doi: 10.1007/s10900-020-00826-8 PMC7725370 32323173

[40] Pietzner M Stewart ID Raffler J Khaw KT Michelotti GA Kastenmuller G. Plasma metabolites to profile pathways in noncommunicable disease multimorbidity. Nat Med. (2021) 27:471–9. doi: 10.1038/s41591-021-01266-0 PMC8127079 33707775

[41] Licher S Heshmatollah A van der Willik KD Stricker BHC Ruiter R de Roos EW. Lifetime risk and multimorbidity of non-communicable diseases and disease-free life expectancy in the general population: A population-based cohort study. PLoS Med. (2019) 16:e1002741. doi: 10.1371/journal.pmed.1002741 30716101 PMC6361416

[42] Graff RE Cavazos TB Thai KK Kachuri L Rashkin SR Hoffman JD. Cross-cancer evaluation of polygenic risk scores for 16 cancer types in two large cohorts. Nat Commun. (2021) 12:970. doi: 10.1038/s41467-021-21288-z 33579919 PMC7880989

[43] Zhang YD Hurson AN Zhang H Choudhury PP Easton DF Milne RL. Assessment of polygenic architecture and risk prediction based on common variants across fourteen cancers. Nat Commun. (2020) 11:3353. doi: 10.1038/s41467-020-16483-3 32620889 PMC7335068

[44] Fritsche LG Gruber SB Wu Z Schmidt EM Zawistowski M Moser SE. Association of polygenic risk scores for multiple cancers in a phenome-wide study: Results from the michigan genomics initiative. Am J Hum Genet. (2018) 102:1048–61. doi: 10.1016/j.ajhg.2018.04.001 PMC5992124 29779563

[45] Davidson KW Barry MJ Mangione CM Cabana M Caughey AB Davis EM. Screening for colorectal cancer: US preventive services task force recommendation statement. Jama. (2021) 325:1965–77. doi: 10.1001/jama.2021.6238 34003218

[46] Grossman DC Curry SJ Owens DK Barry MJ Davidson KW Doubeni CA. Screening for ovarian cancer: US preventive services task force recommendation statement. Jama. (2018) 319:588–94. doi: 10.1001/jama.2017.21926 29450531

[47] Siu AL. Screening for breast cancer: U.S. Preventive services task force recommendation statement. Ann Intern Med. (2016) 164:279–96. doi: 10.7326/M15-2886 26757170

[48] Gierach GL Choudhury PP García-Closas M. Toward risk-stratified breast cancer screening: Considerations for changes in screening guidelines. JAMA Oncol. (2020) 6:31–3. doi: 10.1001/jamaoncol.2019.3820 PMC8170848 31725821

[49] van den Puttelaar R Meester RGS Peterse EFP Zauber AG Zheng J Hayes RB. Risk-stratified screening for colorectal cancer using genetic and environmental risk factors: A cost-effectiveness analysis based on real-world data. Clin Gastroenterol Hepatol. (2023) 21:3415–23.e29. doi: 10.1016/j.cgh.2023.03.003 36906080 PMC10491743

[50] Hull MA Rees CJ Sharp L Koo S. A risk-stratified approach to colorectal cancer prevention and diagnosis. Nat Rev Gastroenterol Hepatol. (2020) 17:773–80. doi: 10.1038/s41575-020-00368-3 PMC7562765 33067592

[51] Wang F Tan F Shen S Wu Z Cao W Yu Y. Risk-stratified approach for never- and ever-smokers in lung cancer screening: A prospective cohort study in China. Am J Respir Crit Care Med. (2023) 207:77–88. doi: 10.1164/rccm.202204-0727OC 35900139

[52] Brentnall AR Cuzick J Buist DSM Bowles EJA. Long-term accuracy of breast cancer risk assessment combining classic risk factors and breast density. JAMA Oncol. (2018) 4:e180174. doi: 10.1001/jamaoncol.2018.0174 29621362 PMC6143016

[53] Kachuri L Graff RE Smith-Byrne K Meyers TJ Rashkin SR Ziv E. Pan-cancer analysis demonstrates that integrating polygenic risk scores with modifiable risk factors improves risk prediction. Nat Commun. (2020) 11:6084. doi: 10.1038/s41467-020-19600-4 33247094 PMC7695829

[54] Hackshaw A Cohen SS Reichert H Kansal AR Chung KC Ofman JJ. Estimating the population health impact of a multi-cancer early detection genomic blood test to complement existing screening in the US and UK. Br J Cancer. (2021) 125:1432–42. doi: 10.1038/s41416-021-01498-4 PMC8575970 34426664

[55] Liu MC Oxnard GR Klein EA Swanton C Seiden MV. Sensitive and specific multi-cancer detection and localization using methylation signatures in cell-free DNA. Ann Oncol. (2020) 31:745–59. doi: 10.1016/j.annonc.2020.04.013 PMC8274402 33506766

[56] Klein EA Richards D Cohn A Tummala M Lapham R Cosgrove D. Clinical validation of a targeted methylation-based multi-cancer early detection test using an independent validation set. Ann Oncol. (2021) 32:1167–77. doi: 10.1016/j.annonc.2021.05.806 34176681

[57] Nadler DL Zurbenko IG. Estimating cancer latency times using a weibull model. Adv Epidemiol. (2014) 2014:746769. doi: 10.1155/2014/746769

[58] Madathil S Rousseau MC Joseph L Coutlée F Schlecht NF Franco E. Latency of tobacco smoking for head and neck cancer among HPV-positive and HPV-negative individuals. Int J Cancer. (2020) 147:56–64. doi: 10.1002/ijc.32708 31584196

[59] Eisenberg MJ Afilalo J Lawler PR Abrahamowicz M Richard H Pilote L. Cancer risk related to low-dose ionizing radiation from cardiac imaging in patients after acute myocardial infarction. Cmaj. (2011) 183:430–6. doi: 10.1503/cmaj.100463 PMC3050947 21324846

[60] Feskanich D Bain C Chan AT Pandeya N Speizer FE Colditz GA. Aspirin and lung cancer risk in a cohort study of women: dosage, duration and latency. Br J Cancer. (2007) 97:1295–9. doi: 10.1038/sj.bjc.6603996 PMC2360462 17895894

