# Supplementary material for: Effects of joint screening for prostate, lung, colorectal, and ovarian cancer – results from a controlled trial
Source: Front Oncol. 2024 Apr 29;14:1322044. doi: 10.3389/fonc.2024.1322044 (PMC11089133; doi:10.3389/fonc.2024.1322044)

**Supplement table 1. Associations between baseline characteristics and male PLCO cancers incidence in the control arm.**

| Characteristics | Subgroups | Non-cancer, n (%) | Cancer, n (%) | Unadjusted | P  value | Adjusted | P  value |
| --- | --- | --- | --- | --- | --- | --- | --- |
|  |  | (N=19144) | (N=3582) | HR (95%CI) |  | HR (95%CI)* |  |
| Age, years | 55-59 | 6483(33.86%) | 811(22.64%) | Ref |  | Ref |  |
|  | 60-64 | 5947(31.06%) | 1218(34.00%) | 1.48(1.35,1.62) | <0.001 | 1.48(1.35,1.62) | <0.001 |
|  | 65-69 | 4212(22.00%) | 1006(28.08%) | 1.76(1.60,1.93) | <0.001 | 1.76(1.60,1.93) | <0.001 |
|  | 70-74 | 2502(13.07%) | 547(15.27%) | 1.77(1.58,1.97) | <0.001 | 1.79(1.60,2.00) | <0.001 |
| Race | White | 16971(88.65%) | 3185(88.92%) | Ref |  |  |  |
|  | Non-white | 2173(11.35%) | 397(11.08%) | 1.02(0.92,1.13) | 0.729 |  |  |
| Smoking | Never | 7093(37.05%) | 1187(33.14%) | Ref |  | Ref |  |
|  | Current | 2165(11.31%) | 507(14.15%) | 1.16(1.08,1.25) | <0.001 | 1.15(1.07,1.24) | <0.001 |
|  | Former | 9886(51.64%) | 1888(52.71%) | 1.54(1.39,1.71) | <0.001 | 1.62(1.46,1.80) | <0.001 |
| BMI, kg/m^2^ | <25 | 5058(26.42%) | 1045(29.17%) | Ref |  |  |  |
|  | 25-29.9 | 9677(50.55%) | 1827(51.01%) | 0.93(0.86,1.00) | 0.052 |  |  |
|  | ≥30 | 4409(23.03%) | 710(19.82%) | 0.84(0.76,0.92) | <0.001 |  |  |
| Aspirin user | No | 9173(47.92%) | 1767(49.33%) | Ref |  |  |  |
|  | Yes | 9971(52.08%) | 1815(50.67%) | 0.97(0.91,1.04) | 0.391 |  |  |
| Ibuprofen user | No | 14790(77.26%) | 2802(78.22%) | Ref |  |  |  |
|  | Yes | 4354(22.74%) | 780(21.78%) | 0.97(0.90,1.05) | 0.478 |  |  |
| Diabetes | No | 17422(91.01%) | 3326(92.85%) | Ref |  | Ref |  |
|  | Yes | 1722(8.99%) | 256(7.15%) | 0.89(0.79,1.01) | 0.077 | 0.86(0.76,0.96) | 0.019 |
| Prostate surgery | None | 17671(92.31%) | 3203(89.42%) | Ref |  | Ref |  |
|  | Full removed | 812(4.24%) | 175(4.89%) | 1.18(1.02,1.38) | 0.031 | 1.03(0.88,1.20) | 0.718 |
|  | Biopsy | 661(3.45%) | 204(5.70%) | 1.62(1.41,1.87) | <0.001 | 1.45(1.25,1.67) | <0.001 |
| Organ-related disease | 0 | 12514(65.37%) | 2181(60.89%) | Ref |  |  |  |
|  | 1 | 4649(24.28%) | 959(26.77%) | 1.15(1.07,1.24) | <0.001 |  |  |
|  | ≥2 | 1981(10.35%) | 442(12.34%) | 1.25(1.13,1.38) | <0.001 |  |  |
| Non-organ-related disease | 0 | 10737(56.09%) | 2050(57.23%) | Ref |  |  |  |
|  | 1 | 6492(33.91%) | 1203(33.58%) | 1.01(0.94,1.01) | 0.742 |  |  |
|  | ≥2 | 1915(10.00%) | 329(9.18%) | 1.04(0.92,1.17) | 0.533 |  |  |
| Screening history | No | 3586(18.73%) | 624(17.42%) | Ref |  |  |  |
|  | Yes | 15558(81.27%) | 2958(82.58%) | 1.05(0.96,1.15) | 0.267 |  |  |
| Family history | No | 14119(73.75%) | 2535(70.77%) | Ref |  | Ref |  |
|  | Yes | 5025(26.25%) | 1047(29.23%) | 1.14(1.06,1.23) | <0.001 | 1.13(1.06,1.22) | <0.001 |

Note: BMI, body mass index. *, variables with P<0.25 in univariate Cox analysis were initially included in multivariable Cox regression analysis.

**Supplement table 2. Associations between baseline characteristics and female PLCO cancers incidence in the control arm.**

| Variable | Subgroups | Non-cancer, n (%) | Cancer, n (%) | Unadjusted | P  value | Adjusted | P  value |
| --- | --- | --- | --- | --- | --- | --- | --- |
|  |  | (N=21943) | (N=840) | HR (95%CI) |  | HR (95%CI)* |  |
| Age, years | 55-59 | 7722(35.14%) | 195(23.21%) | Ref |  | Ref |  |
|  | 60-64 | 6681(30.41%) | 252(30.00%) | 1.39(1.15,1.68) | <0.001 | 1.40(1.16,1.69) | <0.001 |
|  | 65-69 | 4706(21.42%) | 231(27.50%) | 1.83(1.52,2.22) | <0.001 | 1.93(1.58,2.36) | <0.001 |
|  | 70-75 | 2864(13.03%) | 162(19.29%) | 2.20(1.79,2.71) | <0.001 | 2.41(1.93,3.02) | <0.001 |
| Race | White | 19494(88.72%) | 757(90.12%) | Ref |  |  |  |
|  | Non-white | 2479(11.28%) | 83(9.88%) | 0.90(0.92,1.13) | 0.372 |  |  |
| Smoking | Never | 12472(56.76%) | 281(33.45%) | Ref |  | Ref |  |
|  | Current | 1945(8.85%) | 217(25.83%) | 2.01(1.72,2.36) | <0.001 | 2.10(1.79,2.46) | <0.001 |
|  | Former | 7556(34.39%) | 342(40.71%) | 4.96(4.16,5.92) | <0.001 | 5.52(4.61,6.61) | <0.001 |
| BMI, kg/m^2^ | <25 | 9152(41.65%) | 366(43.57%) | Ref |  |  |  |
|  | 25-29.9 | 7558(34.40%) | 286(34.05%) | 0.96(0.82,1.12) | 0.623 |  |  |
|  | ≥30 | 5263(23.95%) | 188(22.38%) | 0.94(0.79,1.12) | 0.500 |  |  |
| Aspirin user | No | 12684(57.73%) | 503(59.88%) | Ref |  |  |  |
|  | Yes | 9289(42.27%) | 337(40.12%) | 0.94(0.82,1.08) | 0.360 |  |  |
| Ibuprofen user | No | 14796(67.34%) | 577(68.69%) | Ref |  |  |  |
|  | Yes | 7177(32.66%) | 263(31.31%) | 0.97(0.84,1.12) | 0.661 |  |  |
| Oral contraception | No | 9804(44.62%) | 446(53.10%) | Ref |  | Ref |  |
|  | Yes | 12155(55.32%) | 393(46.79%) | 0.74(0.64,0.84) | <0.001 | 0.84(0.73,0.98) | 0.022 |
| HRT | No | 7216(32.84%) | 335(39.88%) | Ref |  |  |  |
|  | Yes | 14757(67.16%) | 505(60.12%) | 0.75(0.66,0.87) | <0.001 |  |  |
| Diabetes | No | 20591(93.71%) | 769(91.55%) | Ref |  | Ref |  |
|  | Yes | 1382(6.29%) | 71(8.45%) | 1.50(1.17,1.91) | 0.001 | 1.48(1.16,1.89) | 0.001 |
| Ovary surgery | None | 17605(80.12%) | 711(84.64%) | Ref |  | Ref |  |
|  | Fully removed | 2766(12.59%) | 62(7.38%) | 0.60(0.46,0.78) | <0.001 | 0.59(0.45,0.77) | <0.001 |
|  | Partly removed | 1602(7.29%) | 67(7.98%) | 1.03(0.81,1.32) | 0.816 | 0.89(0.69,1.15) | 0.367 |
| Organ-related disease | 0 | 15781(71.82%) | 550(65.48%) | Ref |  | Ref |  |
|  | 1 | 4954(22.55%) | 214(25.48%) | 1.25(1.06,1.46) | 0.006 | 1.17(0.99,1.37) | 0.056 |
|  | ≥2 | 1238(5.63%) | 76(9.05%) | 1.79(1.41,2.28) | <0.001 | 1.48(1.16,1.89) | 0.002 |
| Non-organ-related disease | 0 | 13624(62.00%) | 523(62.26%) | Ref |  |  |  |
|  | 1 | 7251(33.00%) | 261(31.07%) | 0.97(0.84,1.12) | 0.662 |  |  |
|  | ≥2 | 1098(5.00%) | 56(6.67%) | 1.48(1.12,1.95) | 0.005 |  |  |
| Screening history | No | 6209(28.26%) | 201(23.93%) | Ref |  |  |  |
|  | Yes | 15764(71.74%) | 639(76.07%) | 1.23(1.05,1.44) | 0.012 |  |  |
| Family history | No | 16439(74.81%) | 556(66.19%) | Ref |  | Ref |  |
|  | Yes | 5534(25.19%) | 284(33.81%) | 1.5(1.30,1.73) | <0.001 | 1.40(1.22,1.62) | <0.001 |

Note: BMI, body mass index. HRT, hormone replace therapy. *, variables with P<0.25 in univariate Cox analysis were initially included in multivariable Cox regression analysis.

**Supplementary table 3. Baseline characteristics of male participants between the screening arm and control arm by risk groups.**

| Characteristics | Subgroups | Low risk, n (%) | | |  | High risk, n (%) | | |
| --- | --- | --- | --- | --- | --- | --- | --- | --- |
|  |  | Control arm | Screening arm | P  value |  | Control arm | Screening arm | P  value |
|  |  | (N=12998) | (N=11926) |  |  | (N=19468) | (N=16876) |  |
| Age, years | 55-59 | 9068(69.76%) | 8297(69.57%) | 0.767 |  | 1315(6.75%) | 1232(7.30%) | 0.025 |
|  | 60-64 | 3627(27.90%) | 3371(28.27%) |  |  | 6630(34.06%) | 5805(34.40%) |  |
|  | 65-69 | 174(1.34%) | 146(1.22%) |  |  | 7356(37.79%) | 6410(37.98%) |  |
|  | 70-74 | 129(0.99%) | 112(0.94%) |  |  | 4167(21.40%) | 3429(20.32%) |  |
| Race | White | 11434(87.97%) | 10592(88.81%) | 0.039 |  | 17313(88.93%) | 15047(89.16%) | 0.492 |
|  | Non-white | 1564(12.03%) | 1334(11.19%) |  |  | 2155(11.07%) | 1829(10.84%) |  |
| Smoking  status | Never | 7667(58.99%) | 7126(59.75%) | 0.089 |  | 4154(21.34%) | 3816(22.61%) | 0.001 |
|  | Current | 1646(12.66%) | 1402(11.76%) |  |  | 2179(11.19%) | 1731(10.26%) |  |
|  | Former | 3685(28.35%) | 3398(28.49%) |  |  | 13135(67.47%) | 11329(67.13%) |  |
| BMI, kg/m^2^ | <25 | 3236(24.90%) | 2993(25.10%) | 0.677 |  | 5549(28.50%) | 4661(27.62%) | 0.086 |
|  | 25-29.9 | 6516(50.13%) | 5913(49.58%) |  |  | 9912(50.91%) | 8617(51.06%) |  |
|  | ≥30 | 3246(24.97%) | 3020(25.32%) |  |  | 4007(20.58%) | 3598(21.32%) |  |
| Aspirin user | No | 6555(50.43%) | 6012(50.41%) | 0.985 |  | 9122(46.86%) | 7920(46.93%) | 0.896 |
|  | Yes | 6443(49.57%) | 5914(49.59%) |  |  | 10346(53.14%) | 8956(53.07%) |  |
| Ibuprofen user | No | 9756(75.06%) | 8870(74.38%) | 0.221 |  | 15331(78.75%) | 13231(78.40%) | 0.427 |
|  | Yes | 3242(24.94%) | 3056(25.62%) |  |  | 4137(21.25%) | 3645(21.60%) |  |
| Diabetes | No | 11639(89.54%) | 10781(90.40%) | 0.026 |  | 17974(92.33%) | 15596(92.42%) | 0.764 |
|  | Yes | 1359(10.46%) | 1145(9.60%) |  |  | 1494(7.67%) | 1280(7.58%) |  |
| Prostate surgery | None | 12751(98.10%) | 11733(98.38%) | 0.074 |  | 17046(87.56%) | 15064(89.26%) | <0.001 |
|  | Fully removed | 177(1.36%) | 125(1.05%) |  |  | 1247(6.41%) | 793(4.70%) |  |
|  | Biopsy | 70(0.54%) | 68(0.57%) |  |  | 1175(6.04%) | 1019(6.04%) |  |
| Organ-related disease | 0 | 9572(73.64%) | 8795(73.75%) | 0.932 |  | 11355(58.33%) | 9985(59.17%) | 0.234 |
|  | 1 | 2628(20.22%) | 2391(20.05%) |  |  | 5414(27.81%) | 4626(27.41%) |  |
|  | ≥2 | 798(6.14%) | 740(6.20%) |  |  | 2699(13.86%) | 2265(13.42%) |  |
| Non-organ-related disease | 0 | 7965(61.28%) | 7605(63.77%) | <0.001 |  | 10332(53.07%) | 9183(54.41%) | 0.003 |
|  | 1 | 4098(31.53%) | 3582(30.04%) |  |  | 6876(35.32%) | 5904(34.98%) |  |
|  | ≥2 | 935(7.19%) | 739(6.20%) |  |  | 2260(11.61%) | 1789(10.60%) |  |
| Screening history | No | 2924(22.50%) | 2824(23.68%) | 0.028 |  | 3058(15.71%) | 2811(16.66%) | 0.015 |
|  | Yes | 10074(77.50%) | 9102(76.32%) |  |  | 16410(84.29%) | 14065(83.34%) |  |
| Family history | No | 10716(82.44%) | 9661(81.01%) | 0.004 |  | 13218(67.90%) | 11270(66.78%) | 0.024 |
|  | Yes | 2282(17.56%) | 2265(18.99%) |  |  | 6250(32.10%) | 5606(33.22%) |  |

Note: BMI, body mass index.

**Supplementary table 4. Baseline characteristics of female participants between the screening arm and control arm by risk groups.**

| Characteristics | Subgroups | Low risk, n (%) | | |  | High risk, n (%) | | |
| --- | --- | --- | --- | --- | --- | --- | --- | --- |
|  |  | Control arm | Screening arm | P  value |  | Control arm | Screening arm | P  value |
|  |  | (N=24478) | (N=18928) |  |  | (N=8092) | (N=5742) |  |
| Age, years | 55-59 | 9788(39.99%) | 7727(40.82%) | <0.001 |  | 1444(17.84%) | 1034(25.15%) | 0.044 |
|  | 60-64 | 8000(32.68%) | 6358(33.59%) |  |  | 1899(23.47%) | 1374(33.07%) |  |
|  | 65-69 | 4642(18.96%) | 3410(18.02%) |  |  | 2481(30.66%) | 1845(43.21%) |  |
|  | 70-74 | 2048(8.37%) | 1433(7.57%) |  |  | 2268(28.03%) | 1489(39.50%) |  |
| Race | White | 21833(89.19%) | 16915(89.36%) | 0.580 |  | 7091(87.63%) | 5018(123.49%) | 0.695 |
|  | Non-white | 2645(10.81%) | 2013(10.64%) |  |  | 1001(12.37%) | 724(17.43%) |  |
| Smoking | Never | 17277(70.58%) | 13524(71.45%) | 0.004 |  | 993(12.27%) | 696(17.29%) | 0.383 |
|  | Current | 78(0.32%) | 34(0.18%) |  |  | 3021(37.33%) | 2085(52.61%) |  |
|  | Former | 7123(29.10%) | 5370(28.37%) |  |  | 4078(50.40%) | 2961(71.02%) |  |
| BMI, kg/m^2^ | <25 | 9987(40.80%) | 7605(40.18%) | 0.376 |  | 3491(43.14%) | 2464(60.80%) | 0.643 |
|  | 25-29.9 | 8486(34.67%) | 6596(34.85%) |  |  | 2768(34.21%) | 1939(48.21%) |  |
|  | ≥30 | 6005(24.53%) | 4727(24.97%) |  |  | 1833(22.65%) | 1339(31.92%) |  |
| Aspirin user | No | 14423(58.92%) | 11121(58.75%) | 0.732 |  | 4405(54.44%) | 3155(76.72%) | 0.565 |
|  | Yes | 10055(41.08%) | 7807(41.25%) |  |  | 3687(45.56%) | 2587(64.21%) |  |
| Ibuprofen user | No | 16372(66.88%) | 12720(67.20%) | 0.492 |  | 5576(68.91%) | 3918(97.11%) | 0.411 |
|  | Yes | 8106(33.12%) | 6208(32.80%) |  |  | 2516(31.09%) | 1824(43.82%) |  |
| Oral contraception | No | 9699(39.62%) | 7478(39.51%) | 0.815 |  | 5052(62.43%) | 3579(87.98%) | 0.917 |
|  | Yes | 14779(60.38%) | 11450(60.49%) |  |  | 3040(37.57%) | 2163(52.94%) |  |
| HRT | No | 7355(30.05%) | 5526(29.19%) | 0.055 |  | 3372(41.67%) | 2221(58.73%) | <0.001 |
|  | Yes | 17123(69.95%) | 13402(70.81%) |  |  | 4720(58.33%) | 3521(82.20%) |  |
| Diabetes | No | 23405(95.62%) | 18174(96.02%) | 0.042 |  | 7108(87.84%) | 5045(123.79%) | 0.991 |
|  | Yes | 1073(4.38%) | 754(3.98%) |  |  | 984(12.16%) | 697(17.14%) |  |
| Ovary surgery | None | 19226(78.54%) | 15163(80.11%) | <0.001 |  | 6840(84.53%) | 4908(119.12%) | 0.307 |
|  | Fully removed | 3617(14.78%) | 2624(13.86%) |  |  | 487(6.02%) | 326(8.48%) |  |
|  | Partly removed | 1635(6.68%) | 1141(6.03%) |  |  | 765(9.45%) | 508(13.32%) |  |
| Organ-related disease | 0 | 18626(76.09%) | 14638(77.34%) | 0.008 |  | 4681(57.85%) | 3445(81.52%) | 0.039 |
|  | 1 | 4987(20.37%) | 3677(19.43%) |  |  | 2411(29.79%) | 1632(41.99%) |  |
|  | ≥2 | 865(3.53%) | 613(3.24%) |  |  | 1000(12.36%) | 665(17.42%) |  |
| Non-organ-related disease | 0 | 15485(63.26%) | 12159(64.24%) | 0.103 |  | 4699(58.07%) | 3431(81.84%) | <0.001 |
|  | 1 | 8017(32.75%) | 6022(31.82%) |  |  | 2744(33.91%) | 1954(47.79%) |  |
|  | ≥2 | 976(3.99%) | 747(3.95%) |  |  | 649(8.02%) | 357(11.30%) |  |
| Screening history | No | 7256(29.64%) | 5780(30.54%) | 0.045 |  | 1858(22.96%) | 1291(32.36%) | 0.523 |
|  | Yes | 17222(70.36%) | 13148(69.46%) |  |  | 6234(77.04%) | 4451(108.57%) |  |
| Family history | No | 19312(78.90%) | 14810(78.24%) | 0.103 |  | 4855(60.00%) | 3367(84.55%) | 0.113 |
|  | Yes | 5166(21.10%) | 4118(21.76%) |  |  | 3237(40.00%) | 2375(56.37%) |  |

Note: BMI, body mass index. HRT, hormone replace therapy.

**Supplementary table 5. Baseline characteristics of male participants by compliance.**

|  |  | Group A | Group B | Group C | P value |
| --- | --- | --- | --- | --- | --- |
|  |  | n=14570 | n=17819 | n=1635 |  |
| Age, years | 55-59 | 4787(32.9%) | 5740(32.2%) | 510(31.2%) | <0.001 |
|  | 60-64 | 4762(32.7%) | 5440(30.5%) | 485(29.7%) |  |
|  | 65-69 | 3241(22.2%) | 4212(23.6%) | 375(22.9%) |  |
|  | 70-74 | 1780(12.2%) | 2427(13.6%) | 265(16.2%) |  |
| Race | White | 12997(89.2%) | 15753(88.4%) | 1358(83.1%) | <0.001 |
|  | Non-white | 1573(10.8%) | 2066(11.6%) | 277(16.9%) |  |
| Smoking | Never | 6007(41.2%) | 6103(34.2%) | 475(29.1%) | <0.001 |
| status | Current | 1246(8.55%) | 2395(13.4%) | 320(19.6%) |  |
|  | Former | 7317(50.2%) | 9321(52.3%) | 840(51.4%) |  |
| BMI, kg/m^2^ | <25 | 4066(27.9%) | 4661(26.2%) | 480(29.4%) | <0.001 |
|  | 25-29.9 | 7566(51.9%) | 8677(48.7%) | 790(48.3%) |  |
|  | ≥30 | 2938(20.2%) | 4481(25.1%) | 365(22.3%) |  |
| Aspirin user | No | 7143(49.0%) | 8453(47.4%) | 795(48.6%) | 0.016 |
|  | Yes | 7427(51.0%) | 9366(52.6%) | 840(51.4%) |  |
| Ibuprofen user | No | 11316(77.7%) | 13521(75.9%) | 1255(76.8%) | 0.001 |
|  | Yes | 3254(22.3%) | 4298(24.1%) | 380(23.2%) |  |
| Diabetes | No | 13504(92.7%) | 16083(90.3%) | 1386(84.8%) | <0.001 |
|  | Yes | 1066(7.32%) | 1736(9.74%) | 249(15.2%) |  |
| Organ-related | 0 | 7209(61.7%) | 9128(59.8%) | 878(63.4%) | <0.001 |
| disease | 1 | 3112(26.6%) | 4050(26.5%) | 350(25.3%) |  |
|  | ≥2 | 1366(11.7%) | 2084(13.7%) | 157(11.3%) |  |
| Non-organ | 0 | 8773(60.2%) | 9885(55.5%) | 801(49.0%) | <0.001 |
| related disease | 1 | 4650(31.9%) | 6093(34.2%) | 589(36.0%) |  |
|  | ≥2 | 1147(7.87%) | 1841(10.3%) | 245(15.0%) |  |
| Prostate surgery | None | 13406(92.0%) | 16368(91.9%) | 1524(93.2%) | 0.420 |
|  | Fully removed | 596(4.09%) | 754(4.23%) | 57(3.49%) |  |
|  | Biopsy | 568(3.90%) | 697(3.91%) | 54(3.30%) |  |
| Screening history | No | 2816(19.3%) | 3397(19.1%) | 303(18.5%) | 0.676 |
|  | Yes | 11754(80.7%) | 14422(80.9%) | 1332(81.5%) |  |
| Family history | No | 10700(73.4%) | 12849(72.1%) | 1248(76.3%) | <0.001 |
|  | Yes | 3870(26.6%) | 4970(27.9%) | 387(23.7%) |  |

Note: Group A was defined as complete compliance with the screening protocol. Group B was defined as partial compliance with the screening protocol. Group C was defined as never receiving any screening examinations or tests in the screening arm.

**Supplementary table 6. Baseline characteristics of female participants by compliance.**

|  |  | Group A | Group B | Group C | P value |
| --- | --- | --- | --- | --- | --- |
|  |  | n=8945 | n=21399 | n=2619 |  |
| Age, years | 55-59 | 3221(36.0%) | 7402(34.6%) | 748(28.6%) | <0.001 |
|  | 60-64 | 2959(33.1%) | 6359(29.7%) | 746(28.5%) |  |
|  | 65-69 | 1841(20.6%) | 4717(22.0%) | 637(24.3%) |  |
|  | 70-74 | 924(10.3%) | 2921(13.7%) | 488(18.6%) |  |
| Race | White | 7923(88.6%) | 19109(89.3%) | 2284(87.2%) | <0.001 |
|  | Non-white | 1022(11.4%) | 2290(10.7%) | 335(12.8%) |  |
| Smoking | Never | 5414(60.5%) | 11881(55.5%) | 1287(49.1%) | <0.001 |
| status | Current | 613(6.85%) | 2138(9.99%) | 380(14.5%) |  |
|  | Former | 2918(32.6%) | 7380(34.5%) | 952(36.3%) |  |
| BMI, kg/m^2^ | <25 | 3680(41.1%) | 8785(41.1%) | 1080(41.2%) | 0.002 |
|  | 25-29.9 | 3165(35.4%) | 7329(34.2%) | 837(32.0%) |  |
|  | ≥30 | 2100(23.5%) | 5285(24.7%) | 702(26.8%) |  |
| Aspirin user | No | 5354(59.9%) | 12130(56.7%) | 1468(56.1%) | <0.001 |
|  | Yes | 3591(40.1%) | 9269(43.3%) | 1151(43.9%) |  |
| Ibuprofen user | No | 6218(69.5%) | 14173(66.2%) | 1835(70.1%) | <0.001 |
|  | Yes | 2727(30.5%) | 7226(33.8%) | 784(29.9%) |  |
| Diabetes | No | 8494(95.0%) | 20037(93.6%) | 2370(90.5%) | <0.001 |
|  | Yes | 451(5.04%) | 1362(6.36%) | 249(9.51%) |  |
| Organ-related | 0 | 6988(78.1%) | 14774(69.0%) | 1857(70.9%) | <0.001 |
| disease | 1 | 1646(18.4%) | 5199(24.3%) | 592(22.6%) |  |
|  | ≥2 | 311(3.48%) | 1426(6.66%) | 170(6.49%) |  |
| Non-organ- | 0 | 5916(66.1%) | 13042(60.9%) | 1314(50.2%) | <0.001 |
| related disease | 1 | 2736(30.6%) | 7205(33.7%) | 1080(41.2%) |  |
|  | ≥2 | 293(3.28%) | 1152(5.38%) | 225(8.59%) |  |
| Ovarian surgery | None | 8304(92.8%) | 16047(75.0%) | 2083(79.5%) | <0.001 |
|  | Fully removed | 7(0.08%) | 3831(17.9%) | 332(12.7%) |  |
|  | Partly removed | 634(7.09%) | 1521(7.11%) | 204(7.79%) |  |
| Screening | No | 2816(19.3%) | 3397(19.1%) | 303(18.5%) | 0.676 |
| history | Yes | 11754(80.7%) | 14422(80.9%) | 1332(81.5%) |  |
| Family history | No | 10700(73.4%) | 12849(72.1%) | 1248(76.3%) | <0.001 |
|  | Yes | 3870(26.6%) | 4970(27.9%) | 387(23.7%) |  |
| HRT | No | 3156(35.3%) | 6427(30.0%) | 1156(44.1%) | <0.001 |
|  | Yes | 5789(64.7%) | 14972(70.0%) | 1463(55.9%) |  |
| Oral | No | 8562(95.7%) | 20036(93.6%) | 2420(92.4%) | <0.001 |
| contraception | Yes | 383(4.28%) | 1363(6.37%) | 199(7.60%) |  |

Note: Group A was defined as complete compliance with the screening protocol. Group B was defined as partial compliance with the screening protocol. Group C was defined as never receiving any screening examinations or tests in the screening arm

**Supplementary figure 1. Flowchart of participants’ selection.**


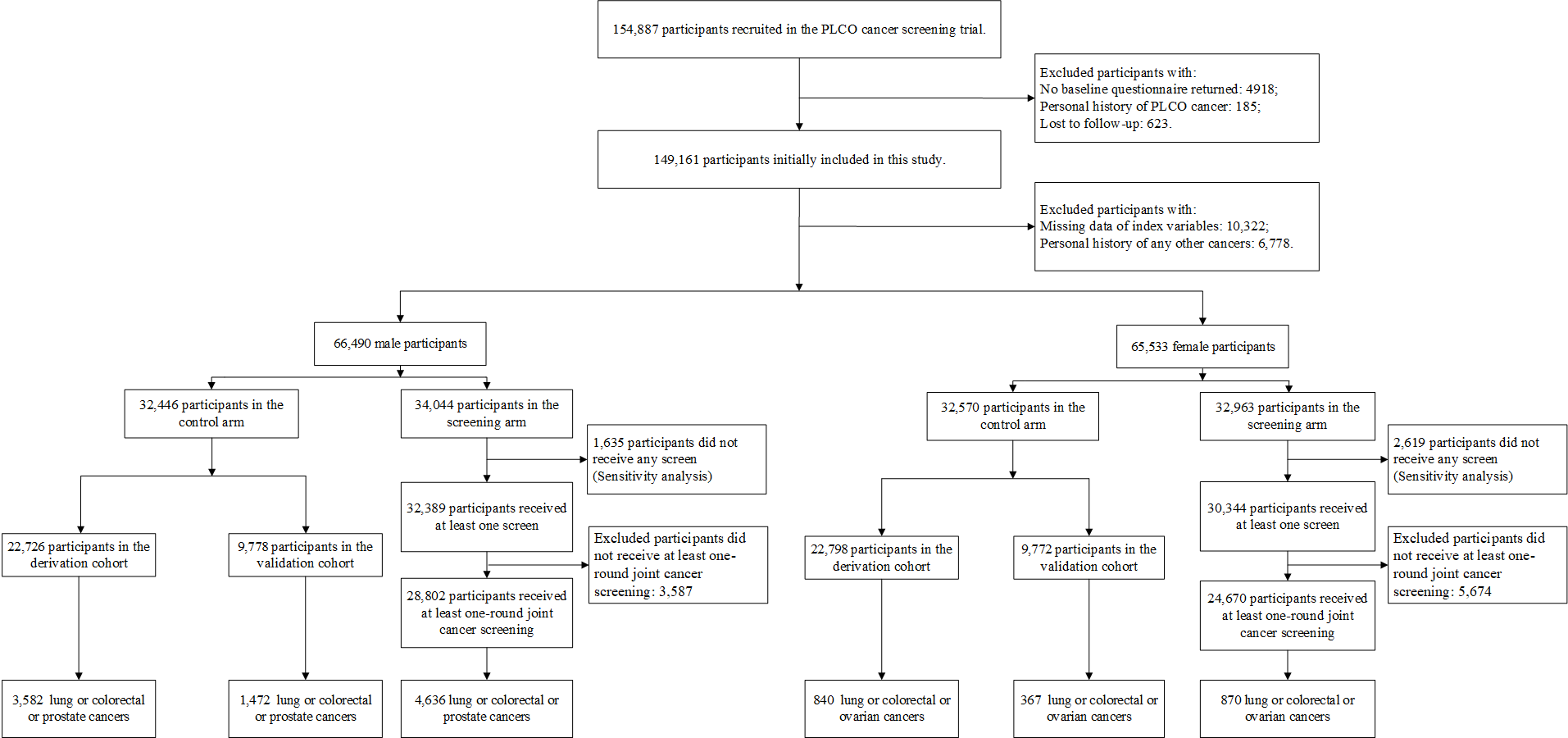


**Supplementary figure 2. CRC-free sensitivity analyses on the effects of joint cancer screening.**


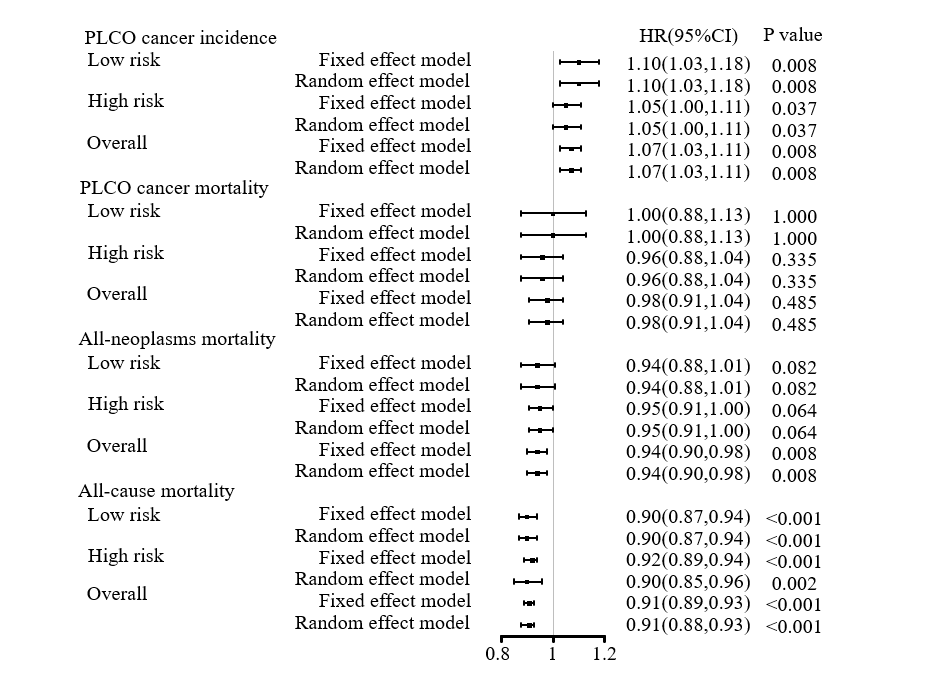


Note: CRC, colorectal cancer.

**Supplementary figure 3. Discriminations and calibration of the gender-specific PLCO-CA models in derivation and validation cohorts.**


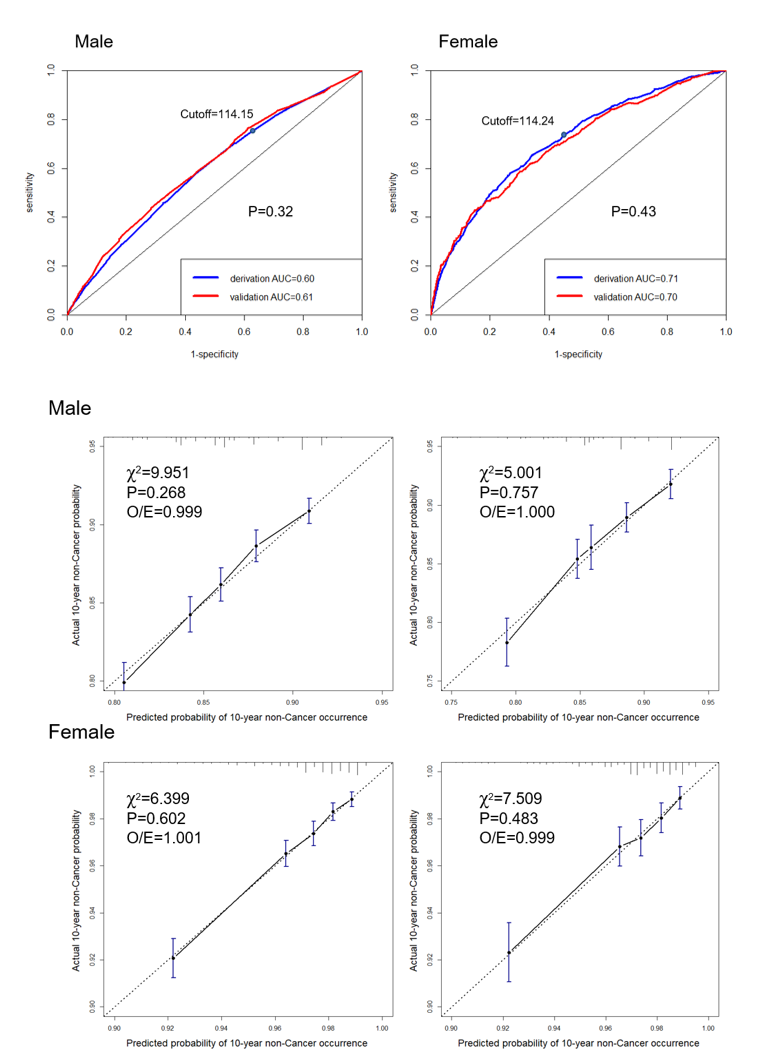


**Supplementary figure 4. Gender-specific effects of joint cancer screening on crude PLCO cancers incidence (A, E), PLCO cancers mortality (B, F), all-neoplasms mortality (C, G), and all-cause mortality (D, H) in low-risk group.**


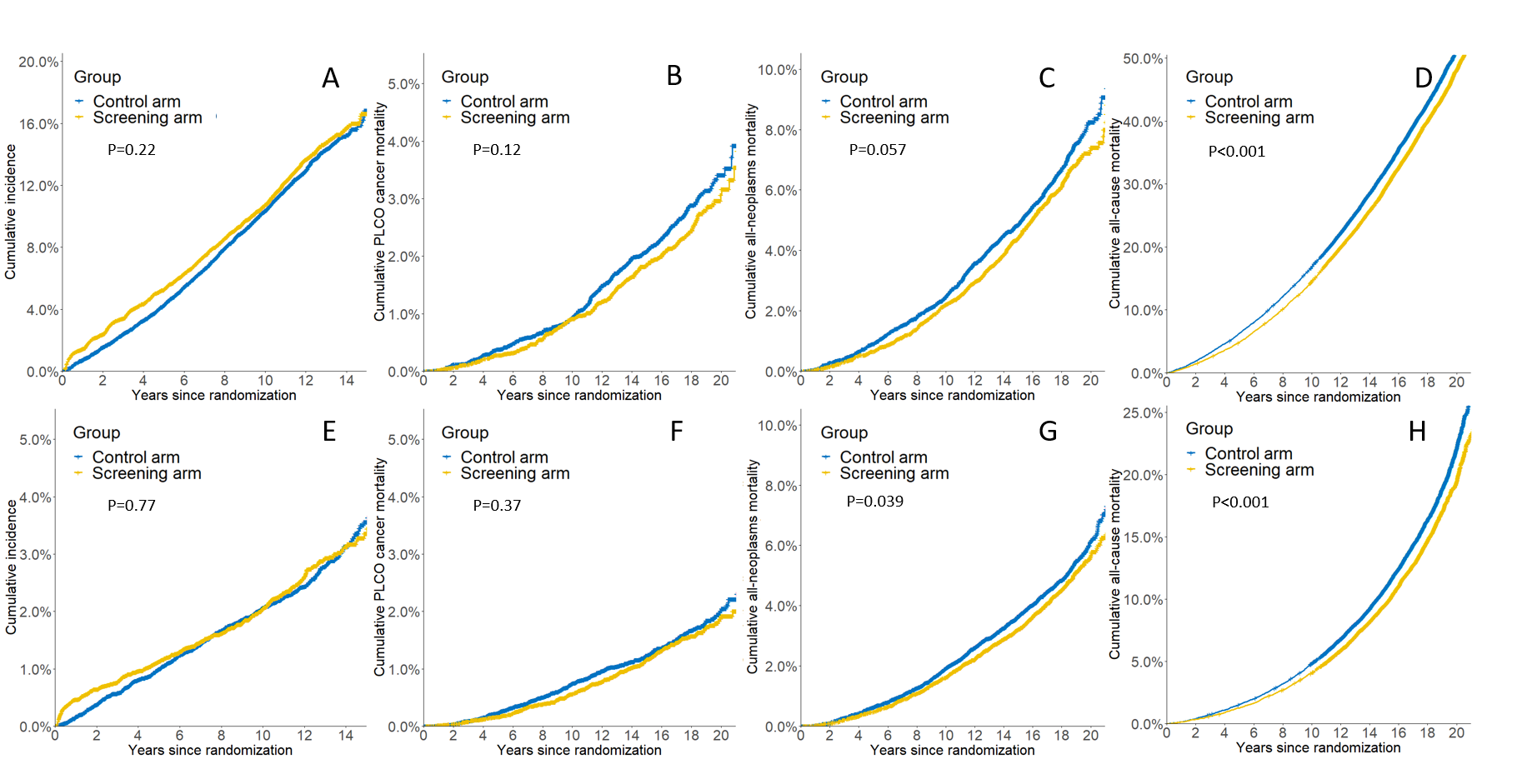


Note: A, B, C, D for males, and E, F, G, H for females.

**Supplementary figure 5. Gender-specific effects of joint cancer screening on crude PLCO cancers incidence (A, E), PLCO cancers mortality (B, F), all-neoplasms mortality (C, G), and all-cause mortality (D, H) in high-risk group.**


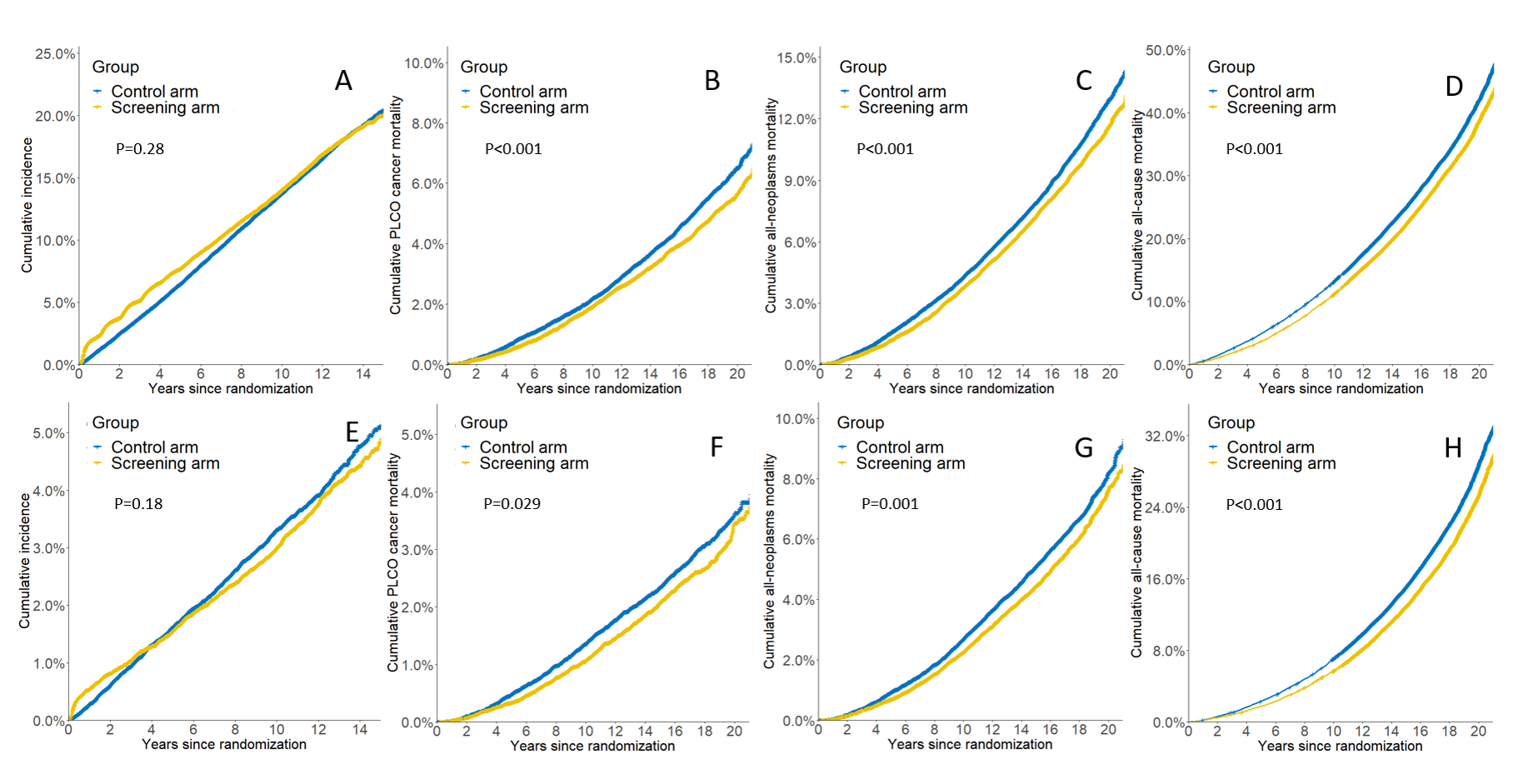


Note: A, B, C, D for males, and E, F, G, H for females.

**Supplementary figure 6. Effects of joint cancer screening for participants with partial compliance to the joint screening protocol.**


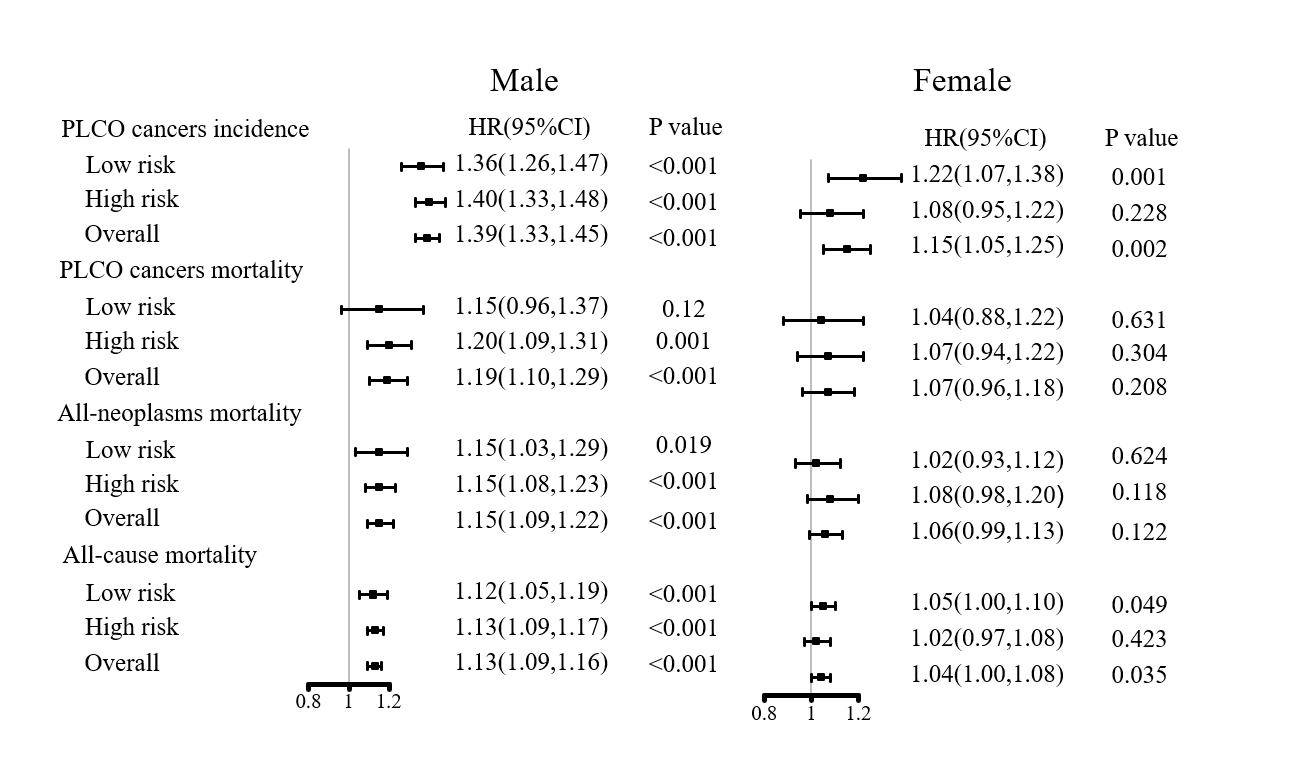

Supplement: Supplementary file 1 [file DataSheet_1.docx]
